# Metagenomic and Resistome Analysis of a Full-Scale Municipal Wastewater Treatment Plant in Singapore Containing Membrane Bioreactors

**DOI:** 10.3389/fmicb.2019.00172

**Published:** 2019-02-18

**Authors:** Charmaine Ng, Boonfei Tan, Xiao-Tao Jiang, Xiaoqiong Gu, Hongjie Chen, Bradley William Schmitz, Laurence Haller, Francis Rathinam Charles, Tong Zhang, Karina Gin

**Affiliations:** ^1^Department of Surgery, National University of Singapore, Singapore, Singapore; ^2^Department of Biological Sciences, University of Alberta, Edmonton, AB, Canada; ^3^Environmental Biotechnology Lab, Department of Civil and Environmental Engineering, The University of Hong Kong, Pokfulam, Hong Kong; ^4^Department of Civil and Environmental Engineering, National University of Singapore, Singapore, Singapore; ^5^JHU/Stantec Alliance, Department of Environmental Health and Engineering, Bloomberg School of Public Health, Johns Hopkins University, Baltimore, MD, United States; ^6^NUS Environmental Research Institute, Singapore, Singapore

**Keywords:** wastewater treatment, antibiotic resistance genes, membrane bioreactor, indicator organisms, metagenomics

## Abstract

Reclaimed water provides a water supply alternative to address problems of scarcity in urbanized cities with high living densities and limited natural water resources. In this study, wastewater metagenomes from 6 stages of a wastewater treatment plant (WWTP) integrating conventional and membrane bioreactor (MBR) treatment were evaluated for diversity of antibiotic resistance genes (ARGs) and bacteria, and relative abundance of class 1 integron integrases (*intl1*). ARGs confering resistance to 12 classes of antibiotics (ARG types) persisted through the treatment stages, which included genes that confer resistance to aminoglycoside *[aadA, aph(6)-I, aph(3*′*)-I, aac(6*′*)-I, aac(6*′*)-II, ant(2*″*)-I]*, beta-lactams [class A, class C, class D beta-lactamases (*bla*_OXA_)], chloramphenicol (acetyltransferase, exporters, *floR, cmIA*), fosmidomycin (*rosAB*), macrolide-lincosamide-streptogramin (*macAB, ereA, ermFB*), multidrug resistance (subunits of transporters), polymyxin (*arnA*), quinolone (*qnrS*), rifamycin (*arr*), sulfonamide (*sul1, sul2*), and tetracycline (*tetM, tetG, tetE, tet36, tet39, tetR, tet43, tetQ, tetX*). Although the ARG subtypes in sludge and MBR effluents reduced in diversity relative to the influent, clinically relevant beta lactamases (i.e., *bla*_KPC_, *bla*_OXA_) were detected, casting light on other potential point sources of ARG dissemination within the wastewater treatment process. To gain a deeper insight into the types of bacteria that may survive the MBR removal process, genome bins were recovered from metagenomic data of MBR effluents. A total of 101 close to complete draft genomes were assembled and annotated to reveal a variety of bacteria bearing metal resistance genes and ARGs in the MBR effluent. Three bins in particular were affiliated to *Mycobacterium smegmatis, Acinetobacter Iwoffii*, and *Flavobacterium psychrophila*, and carried aquired ARGs *aac(2*′*)-Ib, bla*_OXA−278_, and *tet36* respectively. In terms of indicator organisms, cumulative log removal values (LRV) of *Escherichia coli, Enterococci*, and *P. aeruginosa* from influent to conventional treated effluent was lower (0–2.4), compared to MBR effluent (5.3–7.4). We conclude that MBR is an effective treatment method for reducing fecal indicators and ARGs; however, incomplete removal of *P. aeruginosa* in MBR treated effluents (<8 MPN/100 mL) and the presence of ARGs and *intl1* underscores the need to establish if further treatment should be applied prior to reuse.

## Introduction

Wastewater treatment plants (WWTP) receive large volumes of sewage that are enriched in nutrients, chemicals, and bacteria originating from human and animal wastes (Rizzo et al., 2013; Lood et al., 2017). Prevailing resistome datasets point to WWTP as hotspots of antibiotic resistance genes (ARG) and mobile genetic elements (MGE), functioning as point sources for antimicrobial resistance dissemination due to the release of large volumes of treated effluent into the environment (Yang et al., 2014; Ju et al., 2015; Li A. D. et al., 2015; Bengtsson-Palme et al., 2016; Guo et al., 2017). A recent large scale analysis of 484 publically available metagenomes concluded that ARG abundance has strong correlation to fecal pollution (Antti et al., 2018). Hence, it is important to determine if the treatment technology utilized in municipal WWTP is sufficient for removing indicator organisms in effluents at an efficiency that meets public health guidelines to minimize risks, and evaluate the performance of ARG removal. Some studies show that conventional urban WWTP such as suspended-growth processes (activated sludge) have low removal rates of antibiotic resistant bacteria (ARB) and ARGs (Martins da Costa et al., 2006), and in some cases have higher prevalence of antibiotic resistant pathogens in treated effluents (Ferreira da Silva et al., 2007; Luczkiewicz et al., 2010; Al-Jassim et al., 2015) compared to raw influent. This fuels questions regarding the effectiveness of conventional wastewater treatment processes to reduce these emerging contaminants in order to allay public health concerns.

Membrane filtration, although costly, is efficient at removing a high proportion of bacteria due to the association of microbial communities with solid particles in wastewater (Lood et al., 2017). Harb and Hong (2017) reported that membrane bioreactor (MBR) filtering resulted in high log reduction values which is consistent with those in another study (Le et al., 2018). WWTP trains that include a combination of suspended-growth and settling process prior to membrane bioreactor (MBR) treatment are capable of high removal efficiencies (>70%) of certain types of antibiotics (e.g., beta-lactams, glycopeptides, fluoroquinolones) with other antibiotics persisting (e.g., lincomycin, trimethoprim) past MBR treatment (Tran et al., 2016). The exposure of wastewater microbiomes to sub-inhibitory concentrations of antibiotics within different compartments of the wastewater treatment process may allow selection and mutations within the microbial community that enable them to exhibit phenotypic resistance (Chait et al., 2016; Singer et al., 2016). Studies have shown correlations between heavy metals, MGEs [e.g., class 1 integron integrase gene (*intI1*)] and *sul* genes, suggesting that the presence of heavy metals in WWTP play a role in co-selecting for ARGs (Di Cesare et al., 2016). While MBR systems are efficient at removing indicator bacteria such as *Enterococci* and *Escherichia coli* (Francy et al., 2012; Hai et al., 2014), complete removal of all bacteria is still not achieved (Jong et al., 2010; Trinh et al., 2012; van den Akker et al., 2014; Harb and Hong, 2017). It remains a challenge to assess the potential risk associated with reuse of MBR effluents due to lack of information of the types of bacteria that might slip through the treatment process. Furthermore, a recent metagenomic study of resistomes in 4 full-scale water reclamation plants in the United States showed elevated levels of certain ARGs in final effluents (Garner et al., 2018).

The purpose of this study is to examine shifts in the resistome profiles and microbial diversity in a full scale urban WWTP that employs both conventional and MBR for treatment of municipal sewage. Additionally, the removal of indicator organisms was assessed at various stages of the treatment train to determine the performance of each process, and the resultant concentrations of fecal contamination remaining in final effluents. To achieve this, wastewater samples were collected from each stage of the treatment train of a local WWTP during six time points at different intervals over a 12-month timeframe. Utilizing metagenomics sequencing and *in silico* analyses, resistomes and microbial composition of effluents from each sampled stage of the WWTP was profiled to evaluate the types of ARGs that remain after the treatment process.

## Materials and Methods

### Sampling and Concentrations of Microbial Biomass

**Figure 1:**
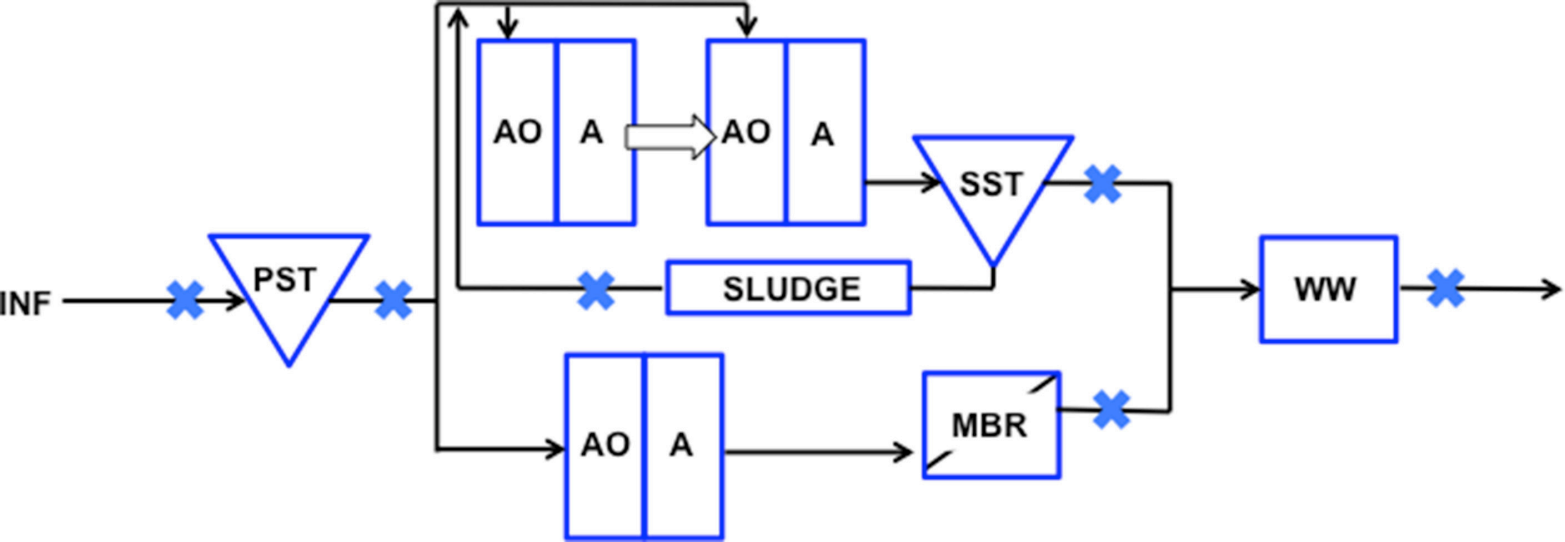
Schematic representation of points sampled along the wastewater reclamation plant. Wastewater was sampled from INF, influent; PST, Primary Settling Tank effluent; SLUDGE, Sludge; SST, Secondary Settling Tank effluent; MBR, Membrane Bioreactor effluent; WW, Wet Well effluent. Sampled points are indicated with a blue cross.

Raw sewage and treated wastewater samples were collected from five locations through the treatment train of a municipal WWTP in Singapore: influent (INF), primary settling tank (PST), secondary settling tank (SST), membrane bioreactor (MBR), and wet well (WW). Also, recycled activated solids (SLUDGE) was collected from a Modified Ludzack-Ettinger (MLE) bioreactor. Samples were collected at six different time points: October 2016, November 2016, January 2017, March 2017, May 2017, August 2017. The water reclamation process and sampling locations are indicated in Figure 1. Wastewater influent is fed into the PST where heavier organic and inorganic matter settle to the bottom and are removed as waste sludge. PST effluent that floats to the surface is pumped into separate trains for biological treatment, a step-feed MLE (anoxic-oxic-anoxic) process prior to being channeled toward SST or MBR treatment. These two points were selected to compare the efficiency in log removal of indicator organisms as a measure of fecal contamination reduction, and determine if adequate removal was achieved. Settled sludge from SST is pumped back into the initial MLE stage as an activated mixed liquor source for biological treatment. The effluent from the SST and MBR trains are then combined in a single storage tank (i.e., wet well) prior to disposal.

Grab samples were collected in sterile Nalgene carboys and initially passed through a filter on-site at the facility prior to further concentration in the laboratory. Turbid samples (INF and PST) were collected in small volumes (1–5 L), while 60 L were collected for clear samples (i.e., SST, MBR, and WW). Recycled SLUDGE (50 mL) was collected in a sterile tube directly from the “return” piping. Samples were concentrated to a final volume of 400 mL using a Hemoflow dialyzer (HF 80s; catalog no. 5007181) via standard bloodline tubing (catalog no. AP16641, Fresenius Medical Care, Bad Homburg, Germany). Prior to concentration, the ultrafilter was rinsed with nanopure water for 5 min, and subsequently pre-treated with 500 mL of blocking solution [0.1 g of Sodium Pyrophosphate (Sigma-Aldrich, Germany) in 1 L of nanopure water] through continuous recirculation for 15 min. After the pre-treatment step, wastewater samples were pumped through the ultrafilter and the retentate tube was blocked. The filtrate was discarded and the biomass trapped on the ultrafilter was eluted with 400 mL of elution buffer [0.1 g of Sodium Pyrophosphate (Sigma-Aldrich, Germany), 5 mL of Tween 80 (Sigma-Aldrich, Germany), 20 μL of antifoam Y-30 (Sigma-Aldrich), 1 L of nanopure water] with the retentate tube unblocked. The concentrates that were eluted from the hollow fiber ultrafiltration unit were then filtered through 0.22 μm cellulose nitrate membrane filters (Sartorius Stedim, Malaysia) to further concentrate the microbial biomass. SLUDGE was centrifuged at 15,000 g for 15 min and the pellet was kept for further analysis.

### Measuring Concentrations of Indicator Organisms

To estimate the level of fecal contamination and determine whether wastewaters met microbiological criteria for recreational purposes (NEA Singapore, 2018), traditional bacterial indicators (*E. coli, Enterococcus* spp., *P. aeruginosa*) were assayed via Colilert, Enterolert, and Pseudalert kits (IDEXX Laboratories Inc., USA) following the manufacturer's instructions with slight modification. Briefly, 100 mL of wastewater sample and 10-fold serially diluted samples were prepared by mixing the respective kit reagents in provided sterile bags and poured into a multi-well tray (Quanti-Tray 2000). All kits were incubated over 24 h at temperatures of 37°C, for Colilert and Pseudalert samples, and 44°C for Enterolert samples. The trays were visualized under long-wave ultraviolet light (365 nm) and positive wells producing a fluorescence was counted and expressed as the most probable number (MPN) per 100 mL of water sample (MPN/100 mL).

### Calculation of Log Removal Values (LRV) for Indicator Organisms

The removal efficiency of each indicator organism through the treatment process was expressed as log removal values (LRV) calculated using the following formula:

$$\begin{array}{l} \text{LRV}={\text{log}}_{10}\left({\text{C}}_{IN}\right)-{\text{log}}_{10}\left({\text{C}}_{OUT}\right) \end{array}$$

where *C*_*IN*_ represents the concentration in the inlet wastewater source and C_*out*_ represents the concentration in the effluent of the treatment stage. For effluent samples that did not contain detectable concentrations of indicators (<1 MPN/100 mL), a “zero” value was substituted in order to calculate LRV representative of the actual removal.

### DNA Extraction

Nucleic acids were extracted from total wastewater biomass concentrated on 0.22 μm cellulose nitrate membrane filters (see section Sampling and Concentrations of Microbial Biomass) and 0.5 g (wet weight) of SLUDGE samples using the PowerSoil DNA Isolation Kit (Qiagen, Netherlands) according to the manufacturer's recommendations. The quality and quantity of DNA extracted were determined using a Qubit 3.0 Fluorometer (Thermo Fisher Scientific, United States) and extracted DNA was run on a 1% agarose gel to ensure that samples were not degraded.

### Library Preparation and Sequencing

Extracted DNA was sent to the Singapore Centre of Environmental Life Sciences and Engineering (SCELSE) where library preparation and sequencing was performed on the Illumina HiSeq2500 sequencer as stated in Ng et al. (2017). Metagenomic datasets were deposited into the NCBI short read archive (SRA) under BioProject accession PRJNA438174 (Table S1).

### Taxonomic Assignment and Identification of ARGs and Integron Genes

All paired end raw reads were removed of sequence adaptors and low quality reads using bbmaps of the BBTools packages (https://sourceforge.net/projects/bbmap/). Taxonomic assignment of clean reads was done using Metaxa2 2.2 beta 10 (Bengtsson-Palme et al., 2015) with the default parameters, and results were analyzed using MEGAN 6 (Huson et al., 2016) for microbial diversity analysis. ARGs-OAP platform (http://smile.hku.hk/SARGs), an online analysis pipeline developed by Yang et al. (2016), was used for antibiotic resistance gene detection through interrogation against A Structured ARG reference database (SARG). SARG was constructed by extracting unique ARG sequences from the commonly used Antibiotic Resistance Database (ARDB) and the Comprehensive Antibiotic Resistance Database (CARD). ARG-like sequences were identified using the pipeline and sorted into subtypes (e.g., *tetA, tetX, tetY*) and types (e.g., tetracycline) with relative abundances measured by normalizing the number of assigned ARG reads to 16S rRNA genes in the metagenomic dataset for each sample. Cleaned reads were interrogated against integron-like sequences extracted from the INTEGRALL database (Moura et al., 2009), and relative abundance was expressed by normalizing the number of integron assigned reads to the number of 16S rRNA genes assigned reads for each sample. A log (x + 1) transformation was applied to integron relative abundance values and an unpaired Student's *t*-test was used to determine statistical differences (*P* < 0.05) between the different treatment steps.

### Statistical Analysis

To construct heatplots of the relative abundance of ARG subtypes and types in each sample, data was normalized using a log (x + 1) transformation and plotted using the online web tool ClustVis (https://biit.cs.ut.ee/clustvis/). Primer v7 (Clarke and Gorley, 2015) was used to analyze clustering patterns of ARGs and microbial community structure (at the genus level). A log (x + 1) transformation was used to normalize datasets and a resemblance matrix was calculated using a Bray-Curtis analysis. Principal Coordinate Analysis (PCO) was used to illustrate clustering patterns of samples and an Analysis of Similarity (ANOSIM) procedure using 999 iterations were used to test for significance of the clustered groups. Only ARGs or microbial taxa that had a Pearson correlation of >0.8 within clustered groups were represented as vectors in PCO plots.

### Metagenomic Binning of MBR Effluent

All clean metagenomics reads from the MBR effluent were assembled into contigs using Megahit (Li D. et al., 2015). Genomic binning was conducted using Metabat 2 (Kang et al., 2015) and MaxBin (Wu et al., 2014), after which genome refinement was done using MetaWRAP (Uritskiy et al., 2018). The quality of genomes recovered was measured for completeness and contamination using CheckM (Parks et al., 2014). To identify genomes containing acquired ARGs, each bin was searched against Resfinder 3.0 (https://cge.cbs.dtu.dk/services/ResFinder/) at a selected threshold of 90% and minimum query length of 60%. Gene calling and functional annotation of genome bins were performed using RAST (Rapid Annotation using Subsystem Technology, Overbeek et al., 2014) available online (http://rast.nmpdr.org/).

## Results

### Removal Efficiency of Indicator Organisms

Although mean concentrations for each indicator were abundant in raw sewage (*E. coli* 1.3 × 10^7^ ± 8.9 × 10^6^ MPN/100 mL, *Enterococci* 1.1 × 10^6^ ± 5.6 × 10^5^, *P. aeruginosa* 4.7 × 10^6^ ± 4.8 × 10^6^ MPN/100 mL), sedimentation during PST stages resulted in only slight reduction (<1.00) of *E. coli* (2.3 × 10^7^ ± 2.3 × 10^7^ MPN/100 mL) and *Enterococci* (6.8 × 10^5^ ± 2.2 × 10^5^ MPN/100 mL), as well as *P. aeruginosa* (4.7 × 10^6^ ± 4.8 × 10^6^ MPN/100 mL) (Table 1). Suspended-growth AO-A bioreactors followed by SST resulted in mean LRV of 1.35 for *E. coli* and 1.77 for *P. aeruginosa*, and 1.81 for *Enterococci*. However, secondary effluent from this train contained 10^4^ to 10^5^ MPN/100 mL of each indicator organism. The majority of indicator removal occurred during MBR secondary treatment, as this stage resulted in mean LRV of 6.86 ± 0.80 for *E. coli*, 5.98 ± 0.22 for *Enterococci* and 6.03 ± 0.58 for *P. aeruginosa*. Effluent from MBR did not contain detectable concentrations of *E. coli* or *Enterococci* (<1 MPN/100 mL), but contained *P. aeruginosa* at a mean concentration of 4.0 ± 2.8 MPN/100 mL.

**Table 1:** Log removal values (LRV) of indicator organisms along the wastewater treatment train.

	**MPN/100 mL**				**Log value (log**_**10**_**)**				**LRV**		
	**INF**	**PST**	**SST**	**MBR**	**INF**	**PST**	**SST**	**MBR**	**INF→PST**	**INF→SST**	**INF→MBR**
***E. coli***											
Oct 2016	1.41 × 10^7^	1.12 × 10^7^	5.28 × 10^5^	<1	7.15	7.05	5.72	0	0.10	1.43	7.15
Nov 2016	1.66 × 10^7^	1.33 × 10^7^	3.87 × 10^5^	<1	7.22	7.12	5.59	0	0.10	1.63	7.22
Jan 2017	1.45 × 10^7^	1.12 × 10^7^	3.13 × 10^5^	<1	7.16	7.05	5.50	0	0.11	1.66	7.16
Mar 2017	8.84 × 10^6^	5.95 × 10^6^	2.70 × 10^5^	<1	6.95	6.77	5.43	0	0.17	1.52	6.95
May 2017	2.70 × 10^7^	6.87 × 10^7^	1.90 × 10^5^	<1	7.43	7.84	5.28	0	−0.41	2.15	7.43
Aug 2017	1.87 × 10^5^	2.42 × 10^7^	3.87 × 10^5^	<1	5.27	7.38	5.59	0	−2.11	−0.32	5.27
***Enterococci***											
Oct 2016	7.27 × 10^5^	7.12 × 10^5^	2.26 × 10^4^	<1	5.86	5.85	4.35	0	0.01	1.51	5.86
Nov 2016	6.38 × 10^5^	6.13 × 10^5^	1.79 × 10^4^	<1	5.80	5.79	4.25	0	0.02	1.55	5.80
Jan 2017	2.01 × 10^6^	9.87 × 10^5^	1.05 × 10^4^	<1	6.30	5.99	4.02	0	0.31	2.28	6.30
Mar 2017	1.41 × 10^6^	7.76 × 10^5^	1.19 × 10^4^	<1	6.15	5.89	4.08	0	0.26	2.07	6.15
May 2017	1.11 × 10^6^	6.83 × 10^5^	1.19 × 10^4^	<1	6.05	5.83	4.07	0	0.21	1.97	6.05
Aug 2017	5.48 × 10^5^	3.26 × 10^5^	1.87 × 10^4^	<1	5.74	5.51	4.27	0	0.23	1.47	5.74
***P. aeruginosa***											
Oct 2016	1.41 × 10^7^	1.55 × 10^6^	5.94 × 10^4^	<1	7.15	6.19	4.77	0	0.96	2.38	7.15
Nov 2016	4.61 × 10^6^	3.45 × 10^6^	7.27 × 10^4^	7.40	6.66	6.54	4.86	0.87	0.13	1.80	5.79
Jan 2017	1.50 × 10^6^	1.99 × 10^6^	4.61 × 10^4^	2.00	6.18	6.30	4.66	0.30	−0.12	1.51	5.88
Mar 2017	1.12 × 10^6^	3.45 × 10^6^	6.69 × 10^4^	2.00	6.05	6.54	4.83	0.30	−0.49	1.22	5.75
May 2017	2.48 × 10^6^	3.26 × 10^6^	3.79 × 10^4^	7.50	6.39	6.51	4.58	0.88	−0.12	1.82	5.52
Aug 2017	4.11 × 10^6^	5.79 × 10^6^	5.48 × 10^4^	3.10	6.61	6.76	4.74	0.49	−0.15	1.88	6.12

*INF, influent; PST, primary settling tank effluent; SST, secondary settling tank effluent; MBR, membrane bioreactor effluent*.

### Occurrence of ARG Types and Subtypes in the Treatment Train

**Figure 2:**
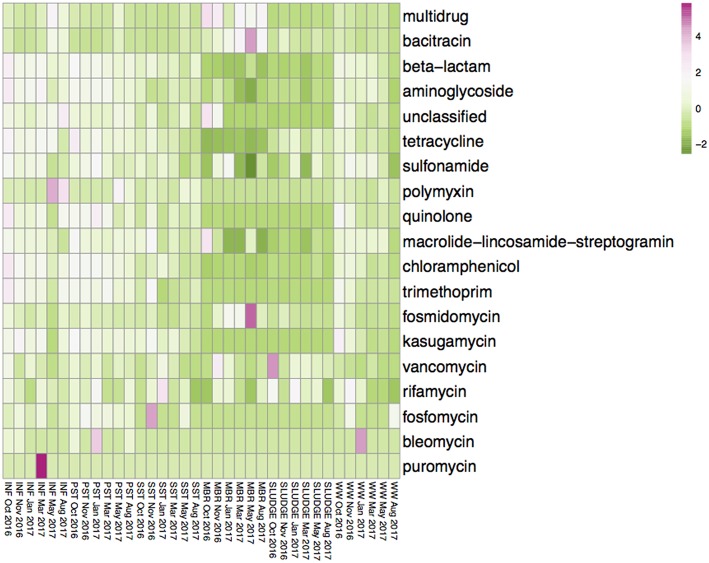
Average values of the relative abundance of ARG subtypes, classified as antibiotic resistance types, in INF, PST, SST, MBR, SLUDGE, and WW samples. Values were transformed using a log (x + 1) function. The color gradient from pink to green represents a higher to lower relative abundance of ARG subtypes assigned to the ARG type.

The average diversity of ARG subtypes was higher in the INF (450 subtypes), PST (460 subtypes), and WW (325 subtypes), compared to the SST (292 subtypes), SLUDGE (195 subtypes), and MBR (181 subtypes) (Table S1). The average normalized abundance of ARG subtypes were highest in MBR (1.350), INF (1.106), and PST (0.857) while the WW, SST, and SLUDGE had lower relative abundances (0.323–0.605) (Table S1). The ARG types with highest relative abundance in INF, PST, and SST were those encoding for multidrug resistance, as well as resistance to beta-lactam and tetracycline antibiotics (Table S2). The MBR, SLUDGE, and WW displayed a slightly different abundance profile. In MBR samples, the dominant ARG types were fosmidomycin, beta-lactam and puromycin; in SLUDGE, tetracycline, multidrug resistance and bacitracin; and in WW, multidrug resistance, beta-lactam and aminoglycoside (Table S2). Across treatment steps, the most dominant ARG subtypes encoded for multidrug resistance and resistance to beta-lactam antibiotics. The 3 most abundant ARG subtypes belonged to the ARG type bacitracin (*bacA*), sulfonamide (*sul1*), aminoglycoside (*aad*), beta-lactam (class A beta-lactamase), multidrug resistance (multidrug transporter, multidrug ABC transporter, *mexF, mexW, mexD, mexI, mexT, adeJ*), tetracycline (*tetX*), and macrolide-lincosamide-streptogramin (MLS, *ermF*) (Figure S1). A heatplot of the ARG types across all samples are represented in Figure 2. ARG subtypes associated with multidrug resistance and resistance to aminoglycoside (*aac, aad, ant, aph*), bacitracin (*bacA*), beta lactams (*bla*_LCR_, *bla*_AER_, *bla*_JOHN_, *bla*_GOB_, *bla*_IMP_, *bla*_OXA_, *bla*_KPC_, *bla*_PDC_, *bla*_PER_), chloramphenicol (chloramphenicol exporters, *cmlA, floR*), fosmidomycin (*rosAB*), MLS (*ere, erm, macAB, msrA*), polymyxin (*arnA*), quinolone (*qepA*), rifamycin (*arr*), sulfonamide (*sul1, sul2*), tetracycline (*tet36, tet39, tet41, tet43, tetA, tetC, tetE, tetG, tetL, tetQ, tetR, tetV, tetX*), trimethoprim (*dfrAB*) and vancomycin (*vanRSX*) were detected in the MBR effluent.

### The Shared Resistome

**Figure 3:**
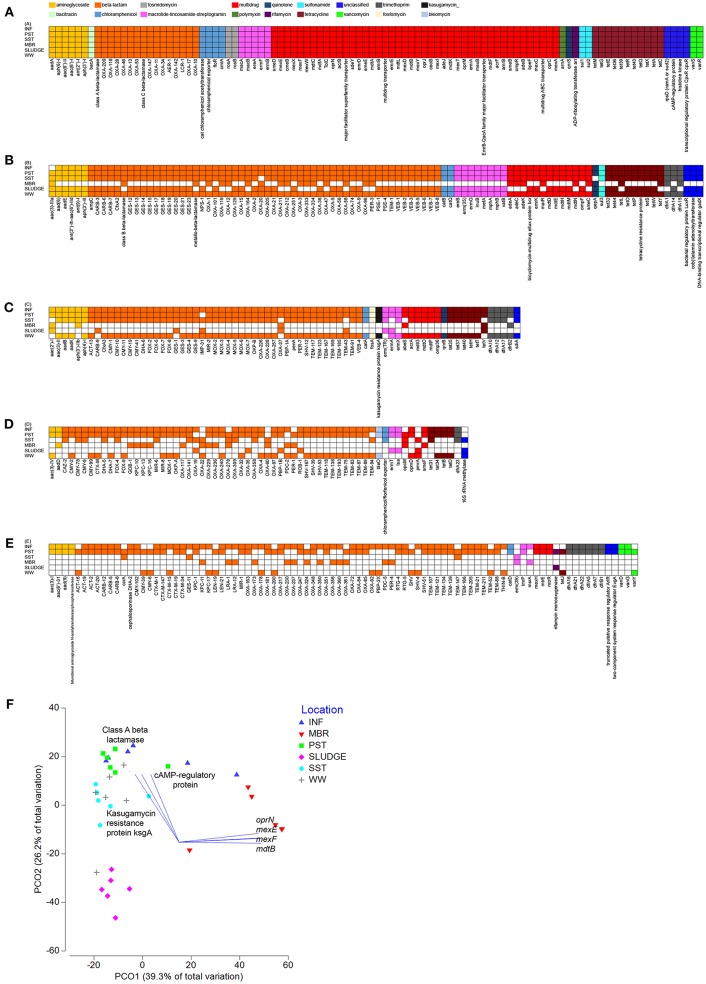
Shared resistomes along the wastewater treatment train. Overlapping ARGs detected at **(A)** all six-, **(B)** five-, **(C)** four-, **(D)** three-, and **(E)** two- treatment stages. The ARG was considered detected as long as it was present in at least one collected time point within the treatment stage. **(F)** A PCO plot of the ARG subtypes. Vectors represent ARGs that had Pearson correlation of >0.8.

Wastewaters from all six locations along the treatment train shared a core resistome of 100 ARG subtypes belonging to 12 ARG types (Figure 3). There were 12 main ARG types that persisted through the treatment stages, which included genes that confer resistance to aminoglycoside [*aadA, aph(6)-I, aph(3*′*)-I, aac(6*′*)-I, aac(6*′*)-II, ant(2*″*)-I*], beta-lactams [class A, class C, class D beta-lactamases (*bla*_OXA_)], chloramphenicol (acetyltransferase, exporters, *floR, cmIA*), fosmidomycin (*rosAB*), MLS (*macAB, ereA, ermFB*), multidrug resistance (subunits of transporters), polymyxin (*arnA*), quinolone (*qnrS*), rifamycin (*arr*), sulfonamide (*sul1, sul2*), and tetracycline (*tetM, tetG, tetE, tet36, tet39, tetR, tet43, tetQ, tetX*) (Figure 3). A Principal Coordinate Analysis (PCO) indicated that at the ARG subtype level, resistome profiles of INF, SST and WW clustered and were different from that of SLUDGE and MBR (ANOSIM test *R* = 0.849, *P* = 0.001, Figure 3). A Pearson correlation (*R* > 0.85, *P* = 0.001) showed that ARG subtypes, class A beta lactamase, cAMP regulatory proteins, and kasugamycin resistance protein (ksgA) correlated with the INF, SST, and WW cluster, while mutidrug efflux pumps (*oprN, mexEF, mdtB*) correlated with the MBR samples.

### Relative Abundance of Class 1 Integron-Integrase Genes (*intI1*)

**Figure 4:**
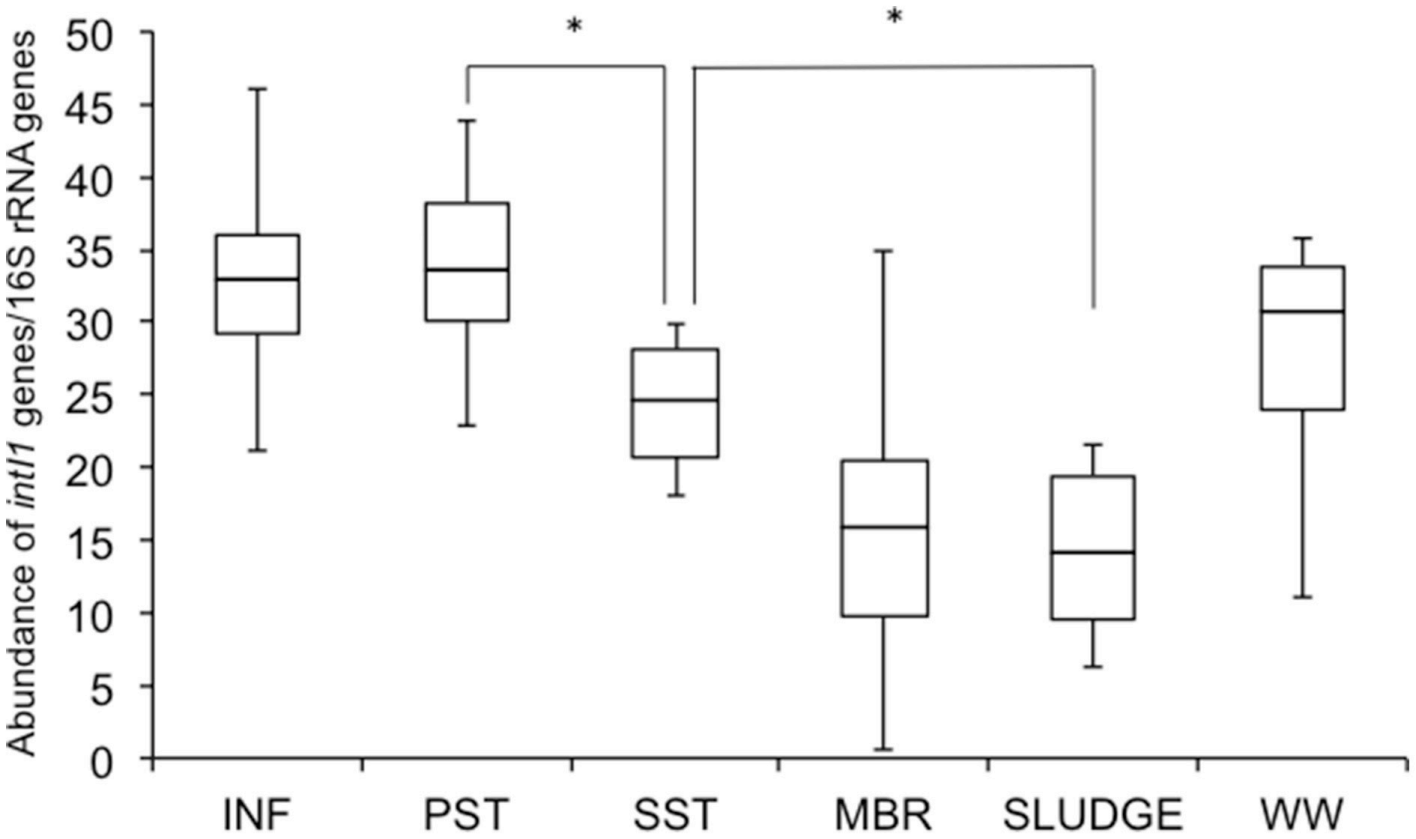
Relative abundance of class 1 integron-integrase genes (intI1) along the wastewater treatment train. Differences were considered significant (^*^) where *P* < 0.05. INF, influent; PST, primary settling tank effluent; SST, secondary settling tank effluent; MBR, membrane bioreactor effluent.

The relative abundance of *intI1* genes was highest in INF (33 ± 9) and PST (34 ± 8), compared to the other points further down the treatment train (WW 28 ± 5, SST 24 ± 5, MBR 16 ± 13, SLUDGE 14 ± 7) (Figure 4). There were no significant differences from the INF to PST, however there was a significant (*P* < 0.05) reduction in relative abundance values from PST to SST, and SST to SLUDGE (Figure 4). There were no significant reductions in the relative abundance of *intI1* genes from SST to MBR.

### Taxonomic Composition

**Figure 5:**
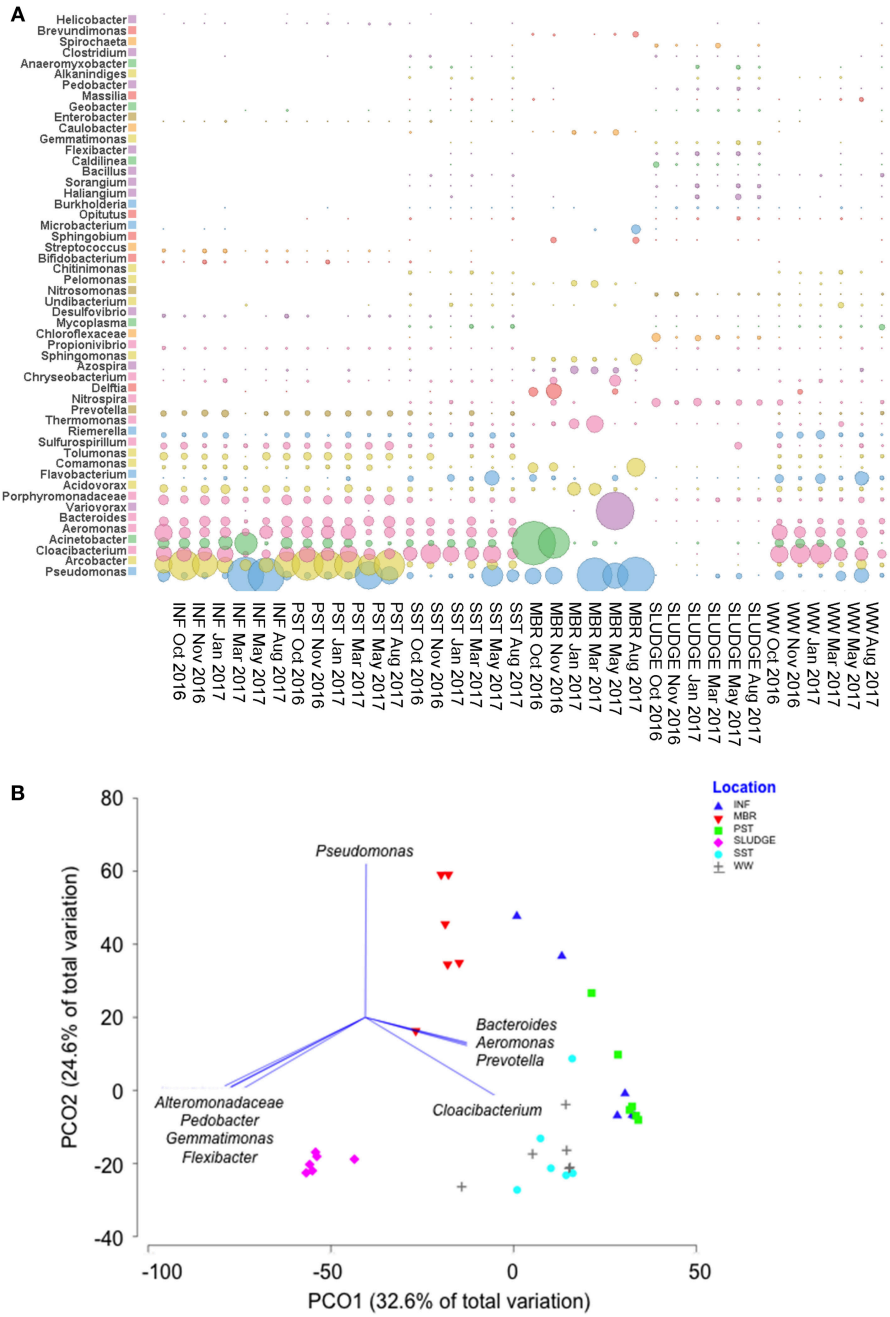
Genera of bacteria detected along the wastewater treatment train. **(A)** A comparison of the most abundant family/genera identified across samples and **(B)** A PCO plot of the bacterial community composition. Vectors represent taxa that had Pearson correlation of >0.8.

The Shannon-Weaver and Simpson reciprocal indices were used to assess the microbial diversity for each sample. The average values showed SLUDGE had the greatest bacterial diversity followed by SST, WW, INF, PST, and MBR (Table S3). A Principal Coordinate Analysis showed that bacterial community structure was divided into two main clusters, one consisting of INF, PST, SST, MBR and WW and another represented by SLUDGE samples (ANOSIM test *R* = 0.357, *P* = 0.01, Figure 5). *Pseudomonas, Bacteroides, Aeromonas, Prevotella*, and *Cloacibacterium* dominated the first cluster in wastewater samples. However, a higher prevalence of *Alteromonadaceae, Pedobacter, Gemmatimonas*, and *Flexibacter* differentiated SLUDGE from the rest of the samples (Pearson correlation *R* > 0.8, Figure 5B). A core microbiome present across all samples was composed of *Pseudomonas, Acinetobacter, Cloacibacterium, Acidovorax, Comamonas, Flavobacterium*, and *Azospira*. *Pseudomonas* (33%), *Acinetobacter* (25%), *Varivorax* (12%), *Comamonas* (5%), *Thermomonas* (4%), *Acidovorax* (3%), and *Delftia* (3%) persisted in post-MBR treated effluent, while *Cloacibacterium* (24%), *Pseudomonas* (11%), *Aeromonas* (8%), *Arcobacter* (6%), *Flavobacterium* (5%), *Bacteroides* (5%), *Acinetobacter* (5%) persisted in SST treated effluent (Figure 5A, Table S4). The WW bacterial community comprised of taxa that were present in SST and MBR effluent with a dominance of *Cloacibacterium* (26%), *Pseudomonas* (8%), *Flavobacterium* (7%), *Acinetobacter* (6%), and *Acidovorax* (2%).

### Binned Bacterial Genomes Recovered From MBR Effluent

**Figure 6:**
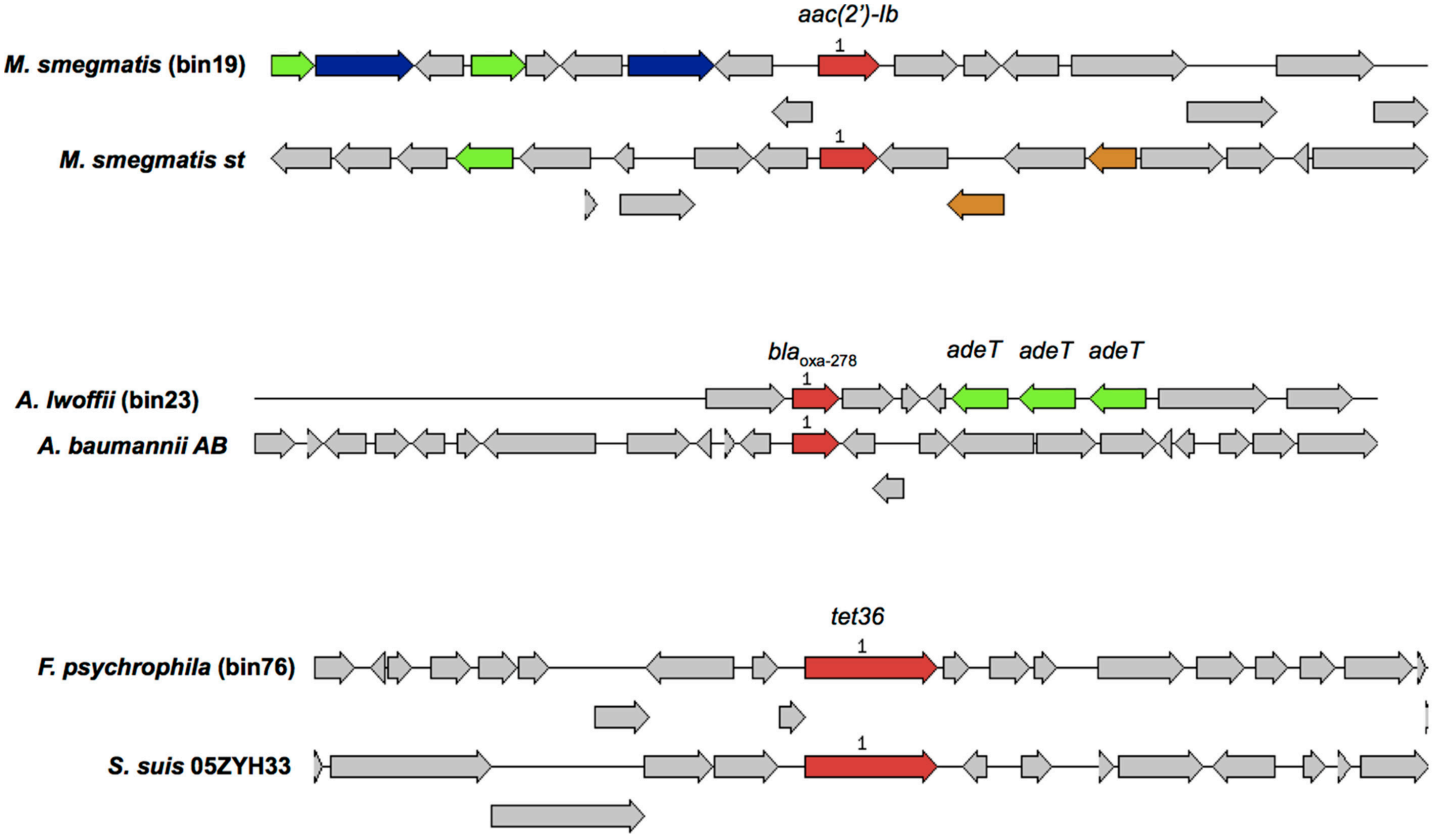
A comparison of binned genomic regions in MBR effluents harboring acquired ARGs. Genomic bin numbers are seen in parenthesis. Sets of genes with similar sequences are grouped by color with those in red representing acquired ARGs identified by Resfinder. Corresponding ARG homologs present in similar organisms were compared against the binned genomic regions.

A total of 101 microbial genomes were recovered and assembled from the MBR treated effluent, and a criteria of >70% completeness and a contamination of <9.6% was selected. The binned genomes were assigned to 59 unique bacterial species based on taxonomic assignments provided by RAST (Table S5). The dominant genera representing at least 3% of the total number of bins included *Magnetospirillum, Acidovorax, Nitrospira, Rhodospirillum, Sphingopyxis, Flavobacterium, Acinetobacteria, Chitinophage, Dechloromonas, Janthinobacterium, Mycobacterium, Novosphingobium* (Table S5). Other potential fish pathogens such as *Flavobacterium* spp., *Chryseobacterium* spp., and a chlamydial bacterial endosymbiont in *Acanthamoebae, Parachlamydiae* spp., and the opportunistic human pathogen *Comomonas* spp. represented at least one genome in the total number of bins identified. Annotation of binned genomes indicated that functional genes related to antibiotic resistance (fluroquinolone resistance, aminoglycoside adenylyltransferase, tetracycline resistance and ribosomal protection type, beta-lactamase, fosfomycin resistance, strepthothricin resistance, multidrug resistance efflux pumps, MexC-MexD-OprJ multidrug efflux systems) and metal resistance (copper homeostasis, cobalt-zinc-cadium resistance, zinc resistance, mercuric reductase/resistance operon, arsenic resistance, chromium compound resistance) were present in the genomes of bacteria in the MBR effluent (Table S6). A query against the Resfinder database identified 3 bins (bins 19, 23, 76) harboring acquired ARGs which showed high homology to *aac(2*′*)-Ib, bla*_OXA−278_, *tet36*, respectively (Figure 6). These three bins were affiliated to *Mycobacterium smegmatis, Acinetobacter Iwoffii, Flavobacterium psychrophila* that carried other genes associated with metal and antibiotic resistance (Table S6).

## Discussion

Water management is one of the cornerstones of sustainable urban development; hence, knowing the efficiency of wastewater treatment technology employed in WWTPs is essential to understand how the effluents may affect downstream processes and the urban water cycle. According to European legislation, traditional parameters such as total suspended solids, chemical oxygen demand, biochemical oxygen demand, ammonia, nitrate, total phosphorus, and fecal coliforms are used to assess the quality of treated wastewaters (EEC, 1998). The indiscriminant usage of antibiotics in human and animals has perpetuated the antimicrobial resistance problem globally (WHO, 2014; Ventola, 2015). Wastewater discharges originating from hospitals and domestic sources carry fecal pathogens, as well as ARB and ARGs which are emerging environmental contaminants (Rizzo et al., 2013; Le et al., 2016; Ng et al., 2017; An et al., 2018; Gupta et al., 2018; Haller et al., 2018; Lorenzo et al., 2018; Manaia et al., 2018). If wastewaters are not treated using appropriate technology, environments receiving wastewater discharge may be impacted (Rodriguez-Mozaz et al., 2015; Proia et al., 2018; Sabri et al., 2018), particularly where fecal indicator concentrations in effluents are high (Antti et al., 2018).

In this full-scale study of a municipal WWTP, cumulative LRV of indicator organisms (*E. coli, Enterococci, P. aeruginosa*) from INF to post-SST was 0–2.4, and 5.3–7.4 post-MBR, which validates a greater removal capacity via MBR as evident in other studies (Harb and Hong, 2017; Zhu et al., 2018). The fecal coliform values were comparable to wastewater treatment plants in Sweden and Italy that apply conventional and MBR treatments (Ottoson et al., 2006). The LRV of *E. coli* reported in our study was slightly higher than that of the Swedish study, as well as LRV median values (5.9) of nine different wastewater MBR systems in California (Hirani et al., 2012). Still, LRV of *E. coli* (7) and *Enterococci* (6) were comparable to another study with a full scale MBR system in a municipal WWTP in Italy (Zanetti et al., 2010). *E. coli* and *Enterococci* in the MBR effluent were below the detection limit (<1MPN/100 mL), which meets the U.S. EPA guidelines for recreational purposes, and within reported limits used for agriculture reuse for surface or spray irrigation of food crop intended for human consumption (U. S. EPA, 1986, 2012).

Despite the absence of *E. coli* and *Enterococci* in MBR effluents, low abundance of viable *P. aeruginosa* were present. The microbial community profile from INF, PST, to SST appear fairly stable with a slight shift observed after MBR treatment, where certain taxa continued to predominated and others waned. The MBR effluent had a microbial community profile dominated by species of *Pseudomonas, Acinetobacter, Varivorax, Comamonas, Thermomonas, Acidovorax, Delftia, Sphingomonas, Chryseobacterium, Azospira*, and *Flavobacteria*. The same genera of bacteria have been described in post MBR treated effluent in several other studies (Grijalbo et al., 2015; Harb and Hong, 2017; Liu et al., 2017). Of these taxa, *Pseudomonas* and *Acinetobacter* are pathogen-associated genera and their detectable levels in MBR treated effluents may pose problems in receiving waters or enable regrowth in storage tanks. Furthermore, the ARG profile of MBR effluents show high relative abundance of fosmidomycin, beta lactam and puromycin ARG types. Also, MBR profiles indicate the persistence of a diversity of 100 ARG subtypes detected throughout the treatment process with resistance to aminoglycoside, beta-lactams (class A, class C, class D beta-lactamases) chloramphenicol, fosmidomycin, MLS, polymyxin, quinolone, rifamycin (*arr*), sulfonamide, tetracycline, and *intI1* genes (associated with horizontal gene transfer). Detection of sulfonamide (*sul1, sul2*), tetracycline (*tetC, tetX, tetG*), MLS (*ereA*) resistant genes and *intI1* in our study has also been observed in MBR effluents of municipal wastewater treatment plants in China (Du et al., 2015; Zhu et al., 2018). Among the beta lactamases, variants of the *bla*_OXA_ genotypes (class D beta lactamase) were detected across the treatment process, including in the sludge. A variety ARG subtypes belonging to 14 different ARG types (multidrug resistance, polymyxin, quinolone, rifamycin, fosmidomycin, chloramphenicol, beta lactam, bacitracin aminoglycoside, sulfonamide, tetracycline, MLS, vancomycin, trimethoprim) were detected in sludge samples which underscores problems of the application of sludge for organic farming purposes. Another study by Calero-Cáceres et al. (2014) provided evidence of sludge being a potentially important source of ARGs.

The ubiquity of different types of *bla*_OXA_ is consistent with previous findings in hospital wastewater effluents and municipal wastewater samples in Singapore (Ng et al., 2017). Variants of class A (*bla*_KPC_, *bla*_PER_), class B (*bla*_GOB_, *bla*_LRA_), and class C (*bla*_PDC_) beta lactamases were detected in INF and MBR effluents, which confer resistance to beta lactam antibiotics such as cephalosporin and carbapenems. These antibiotics are of clinical importance and considered as last resort antibiotics in the treatment of Gram-negative infections (Nordmann et al., 2011; WHO, 2016). This result suggests that even with advanced MBR treatment, these ARG subtypes persist and further treatment and monitoring would be necessary to prevent dissemination. Detection of *bla*_PDC_, a chromosomally inducible *Pseudomonas*-derived cephalosporinase (PDC), and the presence of viable *P. aeruginosa* cells in MBR effluent is a basis for *P.aeruginosa* playing an important role in the carriage and dissemination of antimicrobial resistance in post MBR treated effluent. Although low concentrations of viable *P.aeruginosa* (<8 MPN/100 mL) were measured in the MBR effluent, their biofilm forming ability and antibiotic tolerant properties within a biofilm community (Spoering and Lewis, 2001; Harrison et al., 2005) could allow them to colonize surfaces in the post MBR wastewater treatment train providing a protective environment for other ARB to thrive. Moreover, plasmid bearing *bla*_KPC_ in *P. aeruginosa* strains have been described in local hospital wastewaters (Ng et al., 2017; Haller et al., 2018); providing a basis for prioritizing the monitoring of this ARB in wastewater effluents.

To infer the implications of the detectable array of ARGs in the MBR effluent, metagenomic binning was used to assign ARGs to specific taxa. Only a few binned genomes from the MBR effluent belonged to genera associated with opportunistic pathogens, consistant with taxa (*Acinetobacter, Mycobacterium, Stenotrophomonas, Pseudomonas*) also observed in a 16S rRNA- based microbial classification study of municipal water effluent by Harb and Hong (2017). Two other genome bins affiliated to *Flavobacterium* (*Flavobacterium johnsoniae, Flavobacterium psychrophilum*) and *Chryseobacterium* (*Chryseobacterium gleum*), known to cause infections in fish and occasionally humans (Loch and Faisal, 2015) were recovered from the MBR effluent metagenomes. Metal resistance genes and intrinsic ARGs were annotated in binned bacterial genomes of the MBR effluents. This provides new insights and a direct genetic link of members within the microbial consortia with resistance mechanisms which allow them to protect themselves and survive in the presence of antimicrobials or heavy metals. Only three aquired ARGs were detected in the genome bins of *M. smegmatis, A. Iwoffii, F. psychrophila* and the lack of the identification of more acquired ARGs could be attributed to the difficulties in assigning plasmid sequences to genomic bins. These bacteria are not considered priority pathogens on WHO's published list of antibiotic–resistant “priority pathogens” (Lawe-Davis and Bennett, 2017). Despite this, it should be emphasized that *A. Iwoffii*, which is ubiquitous in the environment is an emerging multidrug resistant pathogen in neonatal sepsis (Mittal et al., 2015). Furthermore, ARGs encoding for resistance against cephalosporins, macrolides, polymyxins, quinolones, and aminoglycosides, that are categorized as critically important antimicrobials for human medicine by WHO (2016), persisted throughout the wastewater treatment process. Monitoring the fate of these genes, beyond the WWTP and into receiving waters, such as the *bla*_OXA_ genes that confer resistance to cephalosporins, could be used to track the extent of dissemination and the evaluate potential risks.

Shifts in community composition were observed at each treatment stage, with the SLUDGE samples having the highest diversity enriched in bacteria involved in the removal of nitrogen (*Nitrospira, Nitromonas)* and phosphorus (*Gemmatimonas*), formation of bioflocs (*Chloroflexaceae, Caldilinea*), and other taxa involved in degradation pathways (*Porphyromonadaceae, Haliangium*) which are typically present in activated sludge (Ferrera and Sanchez, 2016; Shchegolkova et al., 2016) and a good indication of adequate wastewater treatment. Unlike the other samples where beta lactams were the dominant ARG type, the SLUDGE displayed a different profile of tetracycline (*tetX*), bacitracin (*bacA*), and multidrug resistance (*ompR, acrB*) genes.

The reduction in the diversity of ARG subtypes and concentrations of fecal indicators along the treatment train of a full-scale municipal wastewater system is a clear indication that MBR as a treatment strategy is effective at removing raw-sewage associated bacteria and most ARGs in the influent, although removal of *P. aeruginosa* was not as effective. In another study, Zhu et al. (2018) reported that the development of dense membrane fouling layers reinforces the membrane itself creating a dual barrier that prevents leakage of ARGs from the membrane module. Metagenomic binning results indicate that remaining ARGs in the MBR effluent (that were not assigned to any bin) could possibly be extracellular fragmented DNA, plasmid- or phage-associated. The relevance of exogenous ARGs is unknown and it remains a challenge to understand the fate, transport, and health impact that ARGs have on reclaimed water (Hong et al., 2018). Apart from ARG removal, MBR systems have demonstrated higher removal rates of antibiotics and antimicrobials from wastewater influents in contrast to conventional treatment options (Wang et al., 2015; Tran et al., 2016). Although there are currently no benchmarks for maximum admissible values of ARB and ARGs in treated wastewater, our results support the view that MBR is a superior option of treatment for water reuse compared to relying solely on conventional treatment processes that incorporate activated mixed liquors, where effluents still contain relatively high levels of ARGs and indicator organisms. However it should be noted that the MGE, *intI1*, did not show significant rates of removal with MBR treatment.

## Author Contributions

CN, HC, BS, LH, FC, and KG conceptualized and designed the study. CN, HC, BS, LH, and FC processed samples and assisted with data collection. CN, BT, X-TJ, XG, and TZ provided technical expertise in sequence data analysis, assembly, and interpretation. CN and KG wrote the manuscript. KG and BT helped obtain funding support for the work.

### Conflict of Interest Statement

The authors declare that the research was conducted in the absence of any commercial or financial relationships that could be construed as a potential conflict of interest.

## Abbreviations

ARB: antibiotic resistant bacteria
ARG: antibiotic resistant gene
WWTP: wastewater treatment plant
LRV: log removal value
INF: influent
PST: primary settling tank
SST: secondary settling tank
MBR: membrane bioreactor
WW: wet well
SLUDGE: recycle activated sludge
NEA: National Environment Agency
U.S. EPA: United States Environmental Protection Agency.
