# Supplementary material for: Metagenomic and Resistome Analysis of a Full-Scale Municipal Wastewater Treatment Plant in Singapore Containing Membrane Bioreactors
Source: Front Microbiol. 2019 Feb 18;10:172. doi: 10.3389/fmicb.2019.00172 (PMC6387931; doi:10.3389/fmicb.2019.00172)
Supplement: Supplementary file 1 [file Data_Sheet_1.docx]

Supplementary materials for

**Metagenomic and Resistome Analysis of a Full-Scale Municipal Wastewater Treatment Plant in Singapore Containing Membrane Bioreactors**

Charmaine Ng^1^, Boonfei Tan^2^, Xiao-Tao Jiang^3^, Xiaoqiong Gu^4^, Hongjie Chen^4^, Bradley William Schmitz^5^, Laurence Haller^4^, Francis Rathinam Charles^4^, Tong Zhang^3^, Karina Gin^4,6*^

^1^Department of Surgery, National University of Singapore, Singapore

^2^ Department of Biological Sciences, University of Alberta, Edmonton, Alberta, Canada

^3^Environmental Biotechnology Lab, Department of Civil and Environmental Engineering, The University of Hong Kong, Pokfulam, Hong Kong

^4^Department of Civil and Environmental Engineering, National University of Singapore, Singapore, Singapore

^5^ JHU/Stantec Alliance, Department of Environmental Health and Engineering, Bloomberg School of Public Health, Johns Hopkins University, Baltimore, MD, United States

^6^NUS Environmental Research Institute (NERI), Singapore, Singapore

Table S1. Details of metagenomes

| Sample type | Date sampled | Sample ID | Total no. of ARG subtypes | Abundance of ARGs normalised to 16S rRNA genes | No. of unique ARG subtypes identified in sample type | Size of metagenome (GB) | SRA ID |
| --- | --- | --- | --- | --- | --- | --- | --- |
| Influent | October 2016 | INF Oct 2016 | 337 | 1.169 | 450 | 0.217 | SRX3793446 |
|  | November 2016 | INF Nov 2016 | 300 | 0.819 |  | 0.161 | SRX3793444 |
|  | January 2017 | INF Jan 2016 | 299 | 0.802 |  | 0.100 | SRX3793424 |
|  | March 2017 | INF Mar 2017 | 340 | 1.042 |  | 0.107 | SRX3793418 |
|  | May 2017 | INF May 2017 | 257 | 1.682 |  | 0.083 | SRX3793439 |
|  | August 2017 | INF Aug 2017 | 301 | 1.124 |  | 0.188 | SRX3793416 |
| Effluent from Primary Settling Tank (PST) | October 2016 | PST Oct 2016 | 311 | 0.992 | 460 | 0.188 | SRX3793447 |
|  | November 2016 | PST Nov 2016 | 306 | 0.828 |  | 0.154 | SRX3793445 |
|  | January 2017 | PST Jan 2017 | 324 | 0.843 |  | 0.102 | SRX3793425 |
|  | March 2017 | PST Mar 2017 | 325 | 0.764 |  | 0.114 | SRX3793419 |
|  | May 2017 | PST May 2017 | 292 | 0.998 |  | 0.084 | SRX3793438 |
|  | August 2017 | PST Aug 2017 | 307 | 0.715 |  | 0.213 | SRX3793417 |
| Effluent from Secondary Settling Tank (SST) | October 2016 | SST Oct 2016 | 211 | 0.568 | 292 | 0.159 | SRX3793449 |
|  | November 2016 | SST Nov 2016 | 161 | 0.686 |  | 0.153 | SRX3793441 |
|  | January 2017 | SST Jan 2017 | 94 | 0.487 |  | 0.097 | SRX3793423 |
|  | March 2017 | SST Mar 2017 | 198 | 0.545 |  | 0.095 | SRX3793434 |
|  | May 2017 | SST May 2017 | 217 | 0.653 |  | 0.094 | SRX3793436 |
|  | August 2017 | SST Aug 2017 | 176 | 0.524 |  | 0.180 | SRX3793428 |
| Effluent from Membrane Biorector (MBR) | October 2016 | MBR Oct 2016 | 103 | 2.013 | 181 | 0.155 | SRX3793448 |
|  | November 2016 | MBR Nov 2016 | 118 | 1.565 |  | 0.154 | SRX3793440 |
|  | January 2017 | MBR Jan 2017 | 95 | 0.583 |  | 0.100 | SRX3793422 |
|  | March 2017 | MBR Mar 2017 | 99 | 1.261 |  | 0.098 | SRX3793435 |
|  | May 2017 | MBR May 2017 | 63 | 1.336 |  | 0.080 | SRX3793437 |
|  | August 2017 | MBR Aug 2017 | 67 | 1.340 |  | 0.171 | SRX3793431 |
| Sludge (SLUDGE) | October 2016 | SLUDGE Oct 2016 | 117 | 0.284 | 195 | 0.172 | SRX3793443 |
|  | November 2016 | SLUDGE Nov 2016 | 104 | 0.375 |  | 0.181 | SRX3793427 |
|  | January 2017 | SLUDGE Jan 2017 | 106 | 0.374 |  | 0.088 | SRX3793421 |
|  | March 2017 | SLUDGE Mar 2017 | 101 | 0.200 |  | 0.094 | SRX3793432 |
|  | May 2017 | SLUDGE May 2017 | 146 | 0.400 |  | 0.089 | SRX3793429 |
|  | August 2017 | SLUDGE Aug 2017 | 120 | 0.304 |  | 0.222 | SRX3793415 |
| Effluent from Wet Well (WW) | October 2016 | WW Oct 2016 | 272 | 0.893 | 325 | 0.214 | SRX3793442 |
|  | November 2016 | WW Nov 2016 | 201 | 0.774 |  | 0.184 | SRX3793426 |
|  | January 2017 | WW Jan 2017 | 170 | 0.545 |  | 0.098 | SRX3793420 |
|  | March 2017 | WW Mar 2017 | 208 | 0.605 |  | 0.098 | SRX3793433 |
|  | May 2017 | WW May 2017 | 201 | 0.583 |  | 0.086 | SRX3793430 |
|  | August 2017 | WW Aug 2017 | 131 | 0.228 |  | 0.209 | SRX3793414 |

Figure S1. Heatplot of the top 3 most abundant ARG subtypes for each sample. MLS: macrolide-lincosamide-streptogramin.

Table S2. Abundance of ARG types in wastewaters.

|  | INF | PST | SST | MBR | SLUDGE | WW |
| --- | --- | --- | --- | --- | --- | --- |
| aminoglycoside | 0.116 | 0.109 | 0.062 | 0.000 | 0.038 | **0.074** |
| bacitracin | 0.080 | 0.047 | 0.042 | 0.000 | **0.056** | 0.048 |
| beta-lactam | **0.129** | **0.123** | **0.080** | **0.209** | 0.024 | **0.083** |
| bleomycin | 0.000 | 0.000 | 0.000 | 0.000 | 0.000 | 0.000 |
| carbomycin | 0.000 | 0.000 | 0.000 | 0.000 | 0.000 | 0.000 |
| chloramphenicol | 0.019 | 0.019 | 0.009 | 0.000 | 0.003 | 0.009 |
| fosfomycin | 0.000 | 0.001 | 0.000 | 0.001 | 0.000 | 0.000 |
| fosmidomycin | 0.005 | 0.005 | 0.004 | **0.960** | 0.003 | 0.004 |
| fusaric-acid | 0.000 | 0.000 | 0.000 | 0.019 | 0.000 | 0.000 |
| fusidic-acid | 0.000 | 0.000 | 0.000 | 0.000 | 0.000 | 0.000 |
| kasugamycin | 0.003 | 0.003 | 0.002 | 0.000 | 0.000 | 0.001 |
| MLS | 0.051 | 0.055 | 0.045 | 0.000 | 0.017 | 0.044 |
| multidrug | **0.413** | **0.224** | **0.154** | 0.030 | **0.058** | **0.156** |
| polymyxin | 0.015 | 0.008 | 0.006 | 0.000 | 0.001 | 0.004 |
| puromycin | 0.000 | 0.000 | 0.000 | **0.042** | 0.000 | 0.000 |
| quinolone | 0.025 | 0.028 | 0.013 | 0.000 | 0.000 | 0.015 |
| rifamycin | 0.001 | 0.001 | 0.001 | 0.000 | 0.001 | 0.001 |
| spectinomycin | 0.000 | 0.000 | 0.000 | 0.027 | 0.000 | 0.000 |
| sulfonamide | 0.069 | 0.072 | 0.055 | 0.001 | 0.044 | 0.062 |
| tetracenomycin_C | 0.000 | 0.000 | 0.000 | 0.002 | 0.000 | 0.000 |
| tetracycline | **0.121** | **0.116** | **0.074** | 0.018 | **0.073** | 0.072 |
| trimethoprim | 0.008 | 0.008 | 0.004 | 0.030 | 0.000 | 0.004 |
| unclassified | 0.049 | 0.038 | 0.024 | 0.011 | 0.001 | 0.026 |
| vancomycin | 0.002 | 0.002 | 0.001 | 0.000 | 0.004 | 0.000 |

Numbers in bold represent the top 3 most abundant ARG types for each sampled point (by column) along the wastewater treatment train.

Table S3. Bacterial diversity

| Sample | Shannon-Weaver index | Average Shannon-Weaver index values | Simpson reciprocal | Average  Simpson reciprocal |
| --- | --- | --- | --- | --- |
| INF Oct 2016 | 3.644 | 3.034 | 8.678 | 5.456 |
| INF Nov 2016 | 2.817 |  | 3.466 |  |
| INF Jan 2016 | 3.515 |  | 5.545 |  |
| INF Mar 2017 | 4.062 |  | 10.184 |  |
| INF May 2017 | 1.945 |  | 2.417 |  |
| INF Aug 2017 | 2.221 |  | 2.448 |  |
| PST Oct 2016 | 3.339 | 3.026 | 5.533 | 4.362 |
| PST Nov 2016 | 2.789 |  | 3.560 |  |
| PST Jan 2017 | 3.184 |  | 4.341 |  |
| PST Mar 2017 | 3.089 |  | 4.699 |  |
| PST May 2017 | 2.946 |  | 4.195 |  |
| PST Aug 2017 | 2.809 |  | 3.841 |  |
| SST Oct 2016 | 4.960 | 4.240 | 14.656 | 9.854 |
| SST Nov 2016 | 3.802 |  | 5.490 |  |
| SST Jan 2017 | 4.280 |  | 7.621 |  |
| SST Mar 2017 | 4.615 |  | 12.710 |  |
| SST May 2017 | 3.247 |  | 5.912 |  |
| SST Aug 2017 | 4.537 |  | 12.732 |  |
| MBR Oct 2016 | 1.509 | 2.172 | 1.744 | 2.999 |
| MBR Nov 2016 | 2.697 |  | 3.405 |  |
| MBR Jan 2017 | 3.216 |  | 5.971 |  |
| MBR Mar 2017 | 1.880 |  | 2.238 |  |
| MBR May 2017 | 1.807 |  | 2.398 |  |
| MBR Aug 2017 | 1.922 |  | 2.240 |  |
| SLUDGE Oct 2016 | 5.236 | 5.148 | 21.091 | 24.238 |
| SLUDGE Nov 2016 | 5.290 |  | 30.422 |  |
| SLUDGE Jan 2017 | 5.014 |  | 18.441 |  |
| SLUDGE Mar 2017 | 5.225 |  | 27.856 |  |
| SLUDGE May 2017 | 5.003 |  | 22.291 |  |
| SLUDGE Aug 2017 | 5.120 |  | 25.329 |  |
| WW Oct 2016 | 3.815 | 4.020 | 7.931 | 9.818 |
| WW Nov 2016 | 3.540 |  | 5.258 |  |
| WW Jan 2017 | 3.545 |  | 5.386 |  |
| WW Mar 2017 | 4.297 |  | 11.300 |  |
| WW May 2017 | 3.854 |  | 8.960 |  |
| WW Aug 2017 | 5.069 |  | 20.071 |  |

Table S4. Average number of reads assigned to genera. Taxa in asterisk (*) represented the core microbiome

| Genera | INF | PST | SST | MBR | SLUDGE | WW |
| --- | --- | --- | --- | --- | --- | --- |
| *Pseudomonas** | 25.52 | 11.49 | 10.91 | 32.94 | 2.50 | 7.53 |
| *Acinetobacter** | 9.68 | 4.13 | 5.06 | 25.07 | 1.10 | 5.78 |
| *Variovorax* | 0.04 | 0.04 | 0.06 | 12.41 | 0.00 | 0.00 |
| *Comamonas** | 0.97 | 1.09 | 0.71 | 4.65 | 0.47 | 1.01 |
| *Thermomonas* | 0.00 | 0.00 | 0.91 | 4.01 | 0.63 | 1.08 |
| *Acidovorax** | 3.12 | 1.79 | 1.75 | 3.36 | 0.94 | 2.22 |
| *Delftia* | 0.04 | 0.04 | 0.06 | 3.33 | 0.00 | 0.47 |
| *Sphingomonas* | 0.00 | 0.00 | 0.06 | 2.07 | 0.00 | 0.00 |
| *Chryseobacterium* | 0.39 | 0.25 | 0.71 | 1.79 | 0.00 | 0.94 |
| *Azospira** | 0.18 | 0.21 | 0.39 | 1.68 | 0.94 | 0.47 |
| *Flavobacterium** | 0.32 | 0.25 | 5.26 | 1.11 | 0.78 | 7.33 |
| *Pelomonas* | 0.00 | 0.00 | 0.13 | 1.04 | 0.00 | 0.27 |
| *Microbacterium* | 0.11 | 0.00 | 0.00 | 0.86 | 0.16 | 0.00 |
| *Sphingobium* | 0.00 | 0.00 | 0.13 | 0.75 | 0.78 | 0.07 |
| *Brevundimonas* | 0.00 | 0.00 | 0.00 | 0.68 | 0.00 | 0.00 |
| *Caulobacter* | 0.00 | 0.00 | 0.13 | 0.64 | 0.00 | 0.07 |
| *Stenotrophomonas* | 0.07 | 0.04 | 0.00 | 0.54 | 0.00 | 0.00 |
| *Nitrospira* | 0.00 | 0.00 | 0.84 | 0.50 | 9.86 | 1.28 |
| *Sphingopyxis* | 0.00 | 0.00 | 0.00 | 0.39 | 0.00 | 0.00 |
| *Novosphingobium* | 0.00 | 0.00 | 0.13 | 0.32 | 0.47 | 0.13 |
| *Undibacterium* | 0.07 | 0.04 | 0.78 | 0.18 | 0.00 | 1.34 |
| *Rhizobium* | 0.07 | 0.04 | 0.00 | 0.18 | 0.16 | 0.00 |
| *Mycobacterium* | 0.04 | 0.00 | 0.13 | 0.18 | 0.47 | 0.07 |
| *Xanthomonas* | 0.00 | 0.00 | 0.06 | 0.14 | 0.00 | 0.00 |
| *Pseudacidovorax* | 0.00 | 0.00 | 0.00 | 0.14 | 0.00 | 0.00 |
| *Lysobacter* | 0.00 | 0.00 | 0.00 | 0.14 | 0.00 | 0.00 |
| *Massilia* | 0.00 | 0.00 | 0.39 | 0.11 | 0.31 | 0.67 |
| *Sulfurimonas* | 0.00 | 0.00 | 0.13 | 0.11 | 0.00 | 0.07 |
| *Burkholderia* | 0.04 | 0.04 | 0.39 | 0.07 | 1.72 | 0.34 |
| *Sulfuricurvum* | 0.00 | 0.11 | 0.00 | 0.07 | 0.00 | 0.00 |
| *Acidobacteriaceae* | 0.00 | 0.00 | 0.13 | 0.07 | 0.16 | 0.00 |
| *Cloacibacterium** | 8.96 | 10.41 | 23.64 | 0.04 | 0.31 | 25.96 |
| *Rheinheimera* | 0.04 | 0.00 | 0.00 | 0.04 | 0.00 | 0.07 |
| *Legionella* | 0.00 | 0.00 | 0.78 | 0.04 | 0.00 | 0.20 |
| *Phenylobacterium* | 0.00 | 0.00 | 0.45 | 0.04 | 0.63 | 0.34 |
| *Cupriavidus* | 0.00 | 0.00 | 0.39 | 0.04 | 0.63 | 0.20 |
| *Haliangium* | 0.00 | 0.00 | 0.19 | 0.04 | 3.13 | 0.13 |
| *Pedobacter* | 0.00 | 0.00 | 0.13 | 0.04 | 2.35 | 0.20 |
| *Azospirillum* | 0.00 | 0.00 | 0.06 | 0.04 | 0.00 | 0.00 |
| *Methylomonas* | 0.00 | 0.00 | 0.06 | 0.04 | 0.00 | 0.00 |
| *Herbaspirillum* | 0.00 | 0.00 | 0.00 | 0.04 | 0.00 | 0.07 |
| *Dongia* | 0.00 | 0.00 | 0.00 | 0.04 | 0.00 | 0.00 |
| *Ramlibacter* | 0.00 | 0.00 | 0.00 | 0.04 | 0.00 | 0.00 |
| *Curtobacterium* | 0.00 | 0.00 | 0.00 | 0.04 | 0.00 | 0.00 |
| *Arcobacter* | 22.01 | 38.79 | 5.97 | 0.00 | 0.47 | 4.17 |
| *Aeromonas* | 7.85 | 7.22 | 7.60 | 0.00 | 0.00 | 11.77 |
| *Bacteroides* | 4.12 | 5.61 | 5.19 | 0.00 | 0.94 | 3.56 |
| *Porphyromonadaceae* | 3.66 | 4.45 | 2.47 | 0.00 | 3.13 | 1.95 |
| *Tolumonas* | 2.26 | 2.87 | 2.21 | 0.00 | 0.00 | 1.41 |
| *Sulfurospirillum* | 1.76 | 2.84 | 1.30 | 0.00 | 2.19 | 1.28 |
| *Prevotella* | 1.72 | 1.68 | 1.75 | 0.00 | 0.00 | 1.08 |
| *Riemerella* | 1.15 | 1.19 | 2.53 | 0.00 | 0.00 | 3.97 |
| *Streptococcus* | 0.72 | 0.28 | 0.13 | 0.00 | 0.00 | 0.00 |
| *Propionivibrio* | 0.50 | 0.46 | 0.71 | 0.00 | 1.25 | 0.54 |
| *Bifidobacterium* | 0.50 | 0.39 | 0.19 | 0.00 | 0.00 | 0.13 |
| *Desulfovibrio* | 0.47 | 0.42 | 0.52 | 0.00 | 0.94 | 0.34 |
| *Acetoanaerobium* | 0.25 | 0.25 | 0.26 | 0.00 | 0.00 | 0.00 |
| *Enterobacter* | 0.22 | 0.21 | 0.39 | 0.00 | 0.00 | 0.20 |
| *Alistipes* | 0.18 | 0.28 | 0.26 | 0.00 | 0.00 | 0.00 |
| *Synergistaceae* | 0.18 | 0.21 | 0.06 | 0.00 | 0.78 | 0.00 |
| *Desulfomicrobium* | 0.18 | 0.21 | 0.00 | 0.00 | 0.16 | 0.00 |
| *Caminibacter* | 0.18 | 0.21 | 0.00 | 0.00 | 0.00 | 0.00 |
| *Blautia* | 0.18 | 0.04 | 0.00 | 0.00 | 0.00 | 0.00 |
| *Faecalibacterium* | 0.18 | 0.04 | 0.00 | 0.00 | 0.00 | 0.00 |
| *Klebsiella* | 0.14 | 0.18 | 0.19 | 0.00 | 0.00 | 0.20 |
| *Leptotrichia* | 0.14 | 0.18 | 0.00 | 0.00 | 0.00 | 0.00 |
| *Neisseria* | 0.14 | 0.04 | 0.13 | 0.00 | 0.00 | 0.13 |
| *Rhodobacter* | 0.14 | 0.00 | 0.13 | 0.00 | 0.16 | 0.07 |
| *Paracoccus* | 0.14 | 0.00 | 0.00 | 0.00 | 0.94 | 0.00 |
| *Helicobacter* | 0.11 | 0.35 | 0.19 | 0.00 | 0.31 | 0.07 |
| *Lactobacillus* | 0.11 | 0.11 | 0.13 | 0.00 | 0.00 | 0.13 |
| *Shewanella* | 0.11 | 0.11 | 0.00 | 0.00 | 0.00 | 0.00 |
| *Desulfobulbus* | 0.07 | 0.14 | 0.00 | 0.00 | 0.16 | 0.00 |
| *Haemophilus* | 0.07 | 0.14 | 0.00 | 0.00 | 0.00 | 0.00 |
| *Enterococcus* | 0.07 | 0.07 | 0.00 | 0.00 | 0.00 | 0.00 |
| *Fusobacterium* | 0.07 | 0.04 | 0.06 | 0.00 | 0.00 | 0.00 |
| *Roseburia* | 0.07 | 0.04 | 0.00 | 0.00 | 0.00 | 0.00 |
| *Moraxella* | 0.07 | 0.00 | 0.06 | 0.00 | 0.16 | 0.00 |
| *Alicycliphilus* | 0.07 | 0.00 | 0.00 | 0.00 | 0.00 | 0.00 |
| *Brachymonas* | 0.07 | 0.00 | 0.00 | 0.00 | 0.00 | 0.00 |
| *Actinobacillus* | 0.07 | 0.00 | 0.00 | 0.00 | 0.00 | 0.00 |
| *Butyrivibrio* | 0.07 | 0.00 | 0.00 | 0.00 | 0.00 | 0.00 |
| *Subdoligranulum* | 0.07 | 0.00 | 0.00 | 0.00 | 0.00 | 0.00 |
| *Geobacter* | 0.04 | 0.11 | 0.32 | 0.00 | 1.56 | 0.13 |
| *Clostridium* | 0.04 | 0.04 | 0.52 | 0.00 | 1.25 | 0.13 |
| *Pseudoxanthomonas* | 0.04 | 0.04 | 0.00 | 0.00 | 0.00 | 0.00 |
| *Nocardiaceae* | 0.04 | 0.00 | 0.19 | 0.00 | 1.41 | 0.27 |
| *Ralstonia* | 0.04 | 0.00 | 0.06 | 0.00 | 0.00 | 0.07 |
| *Propionibacteriaceae* | 0.04 | 0.00 | 0.00 | 0.00 | 0.16 | 0.00 |
| *Fibrobacter* | 0.04 | 0.00 | 0.00 | 0.00 | 0.00 | 0.00 |
| *Conchiformibius* | 0.04 | 0.00 | 0.00 | 0.00 | 0.00 | 0.00 |
| *Thiothrix* | 0.00 | 0.21 | 0.06 | 0.00 | 0.00 | 0.00 |
| *Magnetospirillum* | 0.00 | 0.14 | 0.13 | 0.00 | 0.00 | 0.00 |
| *Opitutus* | 0.00 | 0.07 | 0.45 | 0.00 | 1.72 | 0.47 |
| *Hydrogenophaga* | 0.00 | 0.04 | 0.39 | 0.00 | 0.00 | 0.61 |
| *Campylobacter* | 0.00 | 0.04 | 0.13 | 0.00 | 0.00 | 0.07 |
| *Vibrionaceae* | 0.00 | 0.04 | 0.06 | 0.00 | 0.00 | 0.00 |
| *Treponema* | 0.00 | 0.04 | 0.00 | 0.00 | 2.19 | 0.00 |
| *Smithella* | 0.00 | 0.04 | 0.00 | 0.00 | 0.94 | 0.00 |
| *Azovibrio* | 0.00 | 0.04 | 0.00 | 0.00 | 0.00 | 0.00 |
| *Desulforegula* | 0.00 | 0.04 | 0.00 | 0.00 | 0.00 | 0.00 |
| *Aquicella* | 0.00 | 0.04 | 0.00 | 0.00 | 0.00 | 0.00 |
| *Pelagicoccus* | 0.00 | 0.04 | 0.00 | 0.00 | 0.00 | 0.00 |
| *Lactococcus* | 0.00 | 0.04 | 0.00 | 0.00 | 0.00 | 0.00 |
| *Caldicoprobacter* | 0.00 | 0.04 | 0.00 | 0.00 | 0.00 | 0.00 |
| *Christensenella* | 0.00 | 0.04 | 0.00 | 0.00 | 0.00 | 0.00 |
| *Anaerostipes* | 0.00 | 0.04 | 0.00 | 0.00 | 0.00 | 0.00 |
| *Coprobacillus* | 0.00 | 0.04 | 0.00 | 0.00 | 0.00 | 0.00 |
| *Mycoplasma* | 0.00 | 0.00 | 1.30 | 0.00 | 0.63 | 1.48 |
| *Chitinimonas* | 0.00 | 0.00 | 1.04 | 0.00 | 0.00 | 1.08 |
| *Alkanindiges* | 0.00 | 0.00 | 0.58 | 0.00 | 0.94 | 0.34 |
| *Bacillus* | 0.00 | 0.00 | 0.58 | 0.00 | 0.78 | 0.67 |
| *Nitrosomonas* | 0.00 | 0.00 | 0.45 | 0.00 | 2.97 | 0.61 |
| *Anaeromyxobacter* | 0.00 | 0.00 | 0.45 | 0.00 | 1.72 | 0.13 |
| *Hippea* | 0.00 | 0.00 | 0.45 | 0.00 | 0.47 | 0.27 |
| *Rickettsia* | 0.00 | 0.00 | 0.39 | 0.00 | 0.47 | 0.40 |
| *Sorangium* | 0.00 | 0.00 | 0.32 | 0.00 | 2.82 | 0.13 |
| *Methylophilaceae* | 0.00 | 0.00 | 0.32 | 0.00 | 0.00 | 0.40 |
| *Planctomyces* | 0.00 | 0.00 | 0.26 | 0.00 | 1.41 | 0.20 |
| *Spiroplasma* | 0.00 | 0.00 | 0.26 | 0.00 | 0.16 | 0.13 |
| *Blastopirellula* | 0.00 | 0.00 | 0.26 | 0.00 | 0.00 | 0.27 |
| *Deinococcus* | 0.00 | 0.00 | 0.26 | 0.00 | 0.00 | 0.27 |
| *Staphylococcus* | 0.00 | 0.00 | 0.26 | 0.00 | 0.00 | 0.27 |
| *Chthoniobacterales* | 0.00 | 0.00 | 0.26 | 0.00 | 0.00 | 0.20 |
| *Flexibacter* | 0.00 | 0.00 | 0.19 | 0.00 | 2.82 | 0.13 |
| *Chlamydiaceae* | 0.00 | 0.00 | 0.19 | 0.00 | 0.00 | 0.20 |
| *Chloroflexaceae* | 0.00 | 0.00 | 0.13 | 0.00 | 6.73 | 0.40 |
| *Gemmatimonas* | 0.00 | 0.00 | 0.13 | 0.00 | 3.13 | 0.07 |
| *Nocardioides* | 0.00 | 0.00 | 0.13 | 0.00 | 0.78 | 0.13 |
| *Pirellula* | 0.00 | 0.00 | 0.13 | 0.00 | 0.16 | 0.13 |
| *Candidatus Phytoplasma* | 0.00 | 0.00 | 0.13 | 0.00 | 0.00 | 0.13 |
| *Meiothermus* | 0.00 | 0.00 | 0.13 | 0.00 | 0.00 | 0.00 |
| *Caldilinea* | 0.00 | 0.00 | 0.06 | 0.00 | 3.13 | 0.13 |
| *Spirochaeta* | 0.00 | 0.00 | 0.06 | 0.00 | 2.82 | 0.07 |
| *Thiobacillus* | 0.00 | 0.00 | 0.06 | 0.00 | 2.03 | 0.00 |
| *Sphingobacterium* | 0.00 | 0.00 | 0.06 | 0.00 | 0.94 | 0.07 |
| *Ottowia* | 0.00 | 0.00 | 0.06 | 0.00 | 0.94 | 0.00 |
| *Cryomorphaceae* | 0.00 | 0.00 | 0.06 | 0.00 | 0.31 | 0.00 |
| *Roseomonas* | 0.00 | 0.00 | 0.06 | 0.00 | 0.31 | 0.00 |
| *Chlorobi* | 0.00 | 0.00 | 0.06 | 0.00 | 0.16 | 0.00 |
| *Victivallis* | 0.00 | 0.00 | 0.06 | 0.00 | 0.16 | 0.00 |
| *Phormidium* | 0.00 | 0.00 | 0.06 | 0.00 | 0.00 | 0.07 |
| *Hydrogenothermus* | 0.00 | 0.00 | 0.06 | 0.00 | 0.00 | 0.00 |
| *Methylobacterium* | 0.00 | 0.00 | 0.06 | 0.00 | 0.00 | 0.00 |
| *Castellaniella* | 0.00 | 0.00 | 0.06 | 0.00 | 0.00 | 0.00 |
| *Limnobacter* | 0.00 | 0.00 | 0.06 | 0.00 | 0.00 | 0.00 |
| *Curvibacter* | 0.00 | 0.00 | 0.06 | 0.00 | 0.00 | 0.00 |
| *Lampropedia* | 0.00 | 0.00 | 0.06 | 0.00 | 0.00 | 0.00 |
| *Pseudorhodoferax* | 0.00 | 0.00 | 0.06 | 0.00 | 0.00 | 0.00 |
| *Simplicispira* | 0.00 | 0.00 | 0.06 | 0.00 | 0.00 | 0.00 |
| *Crenothrix* | 0.00 | 0.00 | 0.06 | 0.00 | 0.00 | 0.00 |
| *Perlucidibaca* | 0.00 | 0.00 | 0.06 | 0.00 | 0.00 | 0.00 |
| *Pseudanabaena* | 0.00 | 0.00 | 0.06 | 0.00 | 0.00 | 0.00 |
| *Thermoactinomycetaceae* | 0.00 | 0.00 | 0.06 | 0.00 | 0.00 | 0.00 |
| *Ruminococcus* | 0.00 | 0.00 | 0.06 | 0.00 | 0.00 | 0.00 |
| *Symbiobacterium* | 0.00 | 0.00 | 0.06 | 0.00 | 0.00 | 0.00 |
| *Alteromonadaceae* | 0.00 | 0.00 | 0.00 | 0.00 | 1.10 | 0.07 |
| *Tetrasphaera* | 0.00 | 0.00 | 0.00 | 0.00 | 0.78 | 0.00 |
| *Longilinea* | 0.00 | 0.00 | 0.00 | 0.00 | 0.78 | 0.00 |
| *Mucilaginibacter* | 0.00 | 0.00 | 0.00 | 0.00 | 0.63 | 0.07 |
| *Hyphomicrobium* | 0.00 | 0.00 | 0.00 | 0.00 | 0.63 | 0.07 |
| *Flexithrix* | 0.00 | 0.00 | 0.00 | 0.00 | 0.63 | 0.00 |
| *Nitrosococcus* | 0.00 | 0.00 | 0.00 | 0.00 | 0.63 | 0.00 |
| *Anaerolinea* | 0.00 | 0.00 | 0.00 | 0.00 | 0.63 | 0.00 |
| *Dehalobacter* | 0.00 | 0.00 | 0.00 | 0.00 | 0.63 | 0.00 |
| *Cyclobacteriaceae* | 0.00 | 0.00 | 0.00 | 0.00 | 0.47 | 0.00 |
| *Rhodopirellula* | 0.00 | 0.00 | 0.00 | 0.00 | 0.47 | 0.00 |
| *Prosthecobacter* | 0.00 | 0.00 | 0.00 | 0.00 | 0.47 | 0.00 |
| *Marmoricola* | 0.00 | 0.00 | 0.00 | 0.00 | 0.47 | 0.00 |
| *Desulfotomaculum* | 0.00 | 0.00 | 0.00 | 0.00 | 0.47 | 0.00 |
| *Syntrophorhabdus* | 0.00 | 0.00 | 0.00 | 0.00 | 0.31 | 0.07 |
| *Prolixibacter* | 0.00 | 0.00 | 0.00 | 0.00 | 0.31 | 0.00 |
| *Spirosoma* | 0.00 | 0.00 | 0.00 | 0.00 | 0.31 | 0.00 |
| *Ectothiorhodospiraceae* | 0.00 | 0.00 | 0.00 | 0.00 | 0.31 | 0.00 |
| *Leptolinea* | 0.00 | 0.00 | 0.00 | 0.00 | 0.31 | 0.00 |
| *Gracilibacter* | 0.00 | 0.00 | 0.00 | 0.00 | 0.31 | 0.00 |
| *Acholeplasma* | 0.00 | 0.00 | 0.00 | 0.00 | 0.31 | 0.00 |
| *Paenibacillus* | 0.00 | 0.00 | 0.00 | 0.00 | 0.16 | 0.13 |
| *Cytophaga* | 0.00 | 0.00 | 0.00 | 0.00 | 0.16 | 0.00 |
| *Afipia* | 0.00 | 0.00 | 0.00 | 0.00 | 0.16 | 0.00 |
| *Nordella* | 0.00 | 0.00 | 0.00 | 0.00 | 0.16 | 0.00 |
| *Bordetella* | 0.00 | 0.00 | 0.00 | 0.00 | 0.16 | 0.00 |
| *Nannocystis* | 0.00 | 0.00 | 0.00 | 0.00 | 0.16 | 0.00 |
| *Chondromyces* | 0.00 | 0.00 | 0.00 | 0.00 | 0.16 | 0.00 |
| *Allochromatium* | 0.00 | 0.00 | 0.00 | 0.00 | 0.16 | 0.00 |
| *Thiococcus* | 0.00 | 0.00 | 0.00 | 0.00 | 0.16 | 0.00 |
| *Methylocaldum* | 0.00 | 0.00 | 0.00 | 0.00 | 0.16 | 0.00 |
| *Phycisphaeraceae* | 0.00 | 0.00 | 0.00 | 0.00 | 0.16 | 0.00 |
| *Luteolibacter* | 0.00 | 0.00 | 0.00 | 0.00 | 0.16 | 0.00 |
| *Actinospica* | 0.00 | 0.00 | 0.00 | 0.00 | 0.16 | 0.00 |
| *Janibacter* | 0.00 | 0.00 | 0.00 | 0.00 | 0.16 | 0.00 |
| *Micromonosporaceae* | 0.00 | 0.00 | 0.00 | 0.00 | 0.16 | 0.00 |
| *Streptomyces* | 0.00 | 0.00 | 0.00 | 0.00 | 0.16 | 0.00 |
| *unclassified Actinobacteria* | 0.00 | 0.00 | 0.00 | 0.00 | 0.16 | 0.00 |
| *Sphaerobacter* | 0.00 | 0.00 | 0.00 | 0.00 | 0.16 | 0.00 |
| *Thermotogaceae* | 0.00 | 0.00 | 0.00 | 0.00 | 0.16 | 0.00 |
| *Schlesneria* | 0.00 | 0.00 | 0.00 | 0.00 | 0.00 | 0.07 |
| *Leucobacter* | 0.00 | 0.00 | 0.00 | 0.00 | 0.00 | 0.07 |
| Total Percentage (%) | 100.00 | 100.00 | 100.00 | 100.00 | 100.00 | 100.00 |

Table S5. Genomic bins of MRB effluent resolved by metaWRAP

| Bin No. | Completeness | Contamination | GC | MetaWRAP lineage | N50 | Size (bp) | Closest neighbour assigned by RAST |
| --- | --- | --- | --- | --- | --- | --- | --- |
| bin.15 | 92.92 | 4.444 | 0.682 | *Bacteria* | 26693 | 3599453 | *Acidobacterium capsulatum ATCC 51196* |
| bin.55 | 76.26 | 1.713 | 0.694 | *Burkholderiales* | 8898 | 3005212 | *Acidovorax avenae subsp. avenae ATCC 19860* |
| bin.29 | 75.59 | 4.413 | 0.693 | *Burkholderiales* | 16172 | 4333741 | *Acidovorax avenae subsp. avenae ATCC 19860* |
| bin.69 | 77.47 | 4.049 | 0.704 | *Burkholderiales* | 9482 | 5442270 | *Acidovorax radicis N35* |
| bin.48 | 88.96 | 5.968 | 0.652 | *Burkholderiales* | 24167 | 4660607 | *Acidovorax radicis N35* |
| bin.70 | 82.69 | 7.071 | 0.705 | *Burkholderiales* | 19955 | 5676545 | *Acidovorax radicis N35* |
| bin.53 | 95.38 | 2.308 | 0.385 | *Moraxellaceae* | 24311 | 2809112 | *Acinetobacter junii SH205* |
| bin.23 | 86.57 | 1.141 | 0.426 | *Moraxellaceae* | 33050 | 2615865 | *Acinetobacter lwoffii SH145* |
| bin.31 | 80.48 | 2.054 | 0.419 | *Moraxellaceae* | 39414 | 2751946 | *Acinetobacter sp. DR1* |
| bin.59 | 74.33 | 4.267 | 0.703 | *Burkholderiales* | 8465 | 3647779 | *Alicycliphilus denitrificans BC* |
| bin.25 | 72.61 | 9.585 | 0.643 | *Alphaproteobacteria* | 4468 | 3298522 | *alpha proteobacterium BAL199* |
| bin.26 | 87.16 | 1.075 | 0.397 | *Bacteria* | 14273 | 1755146 | *Azospirillum sp. B510* |
| bin.5 | 70.42 | 6.425 | 0.662 | *Rhodospirillales* | 6467 | 5328889 | *Azospirillum sp. B510* |
| bin.57 | 84.28 | 5.616 | 0.677 | *Alphaproteobacteria* | 5239 | 2631967 | *Brevundimonas sp. BAL3* |
| bin.101 | 74.81 | 6.034 | 0.685 | *Bacteria* | 7689 | 3562607 | *Brevundimonas sp. BAL3* |
| bin.60 | 72.36 | 9.183 | 0.63 | *Alphaproteobacteria* | 3805 | 3503212 | *Caulobacter sp. K31* |
| bin.87 | 88.62 | 1.395 | 0.489 | *Bacteroidetes* | 8599 | 3508278 | *Chitinophaga pinensis DSM 2588* |
| bin.75 | 98.85 | 1.6 | 0.49 | *Bacteroidetes* | 55553 | 4667984 | *Chitinophaga pinensis DSM 2588* |
| bin.88 | 89.92 | 6.19 | 0.482 | *Bacteroidetes* | 4878 | 3280327 | *Chitinophaga pinensis DSM 2588* |
| bin.39 | 77.83 | 2.046 | 0.636 | *Betaproteobacteria* | 3334 | 2671589 | *Chromobacterium violaceum ATCC 12472* |
| bin.40 | 87.76 | 2.589 | 0.6 | *Betaproteobacteria* | 6901 | 3869149 | *Chromobacterium violaceum ATCC 12472* |
| bin.33 | 100 | 0.49 | 0.359 | *Flavobacteriales* | 107638 | 4309225 | *Chryseobacterium gleum ATCC 35910* |
| bin.11 | 77.55 | 2.45 | 0.663 | *Comamonadaceae* | 4725 | 2697523 | *Comamonas testosteroni KF-1* |
| bin.102 | 84.78 | 3.044 | 0.626 | *Comamonadaceae* | 61459 | 4474311 | *Comamonas testosteroni KF-1* |
| bin.90 | 88.27 | 3.968 | 0.73 | *Actinobacteria* | 6241 | 5264092 | *Conexibacter woesei DSM 14684* |
| bin.17 | 97.59 | 1.05 | 0.68 | *Burkholderiales* | 50670 | 5278498 | *Cupriavidus metallidurans CH34* |
| bin.67 | 74.33 | 4.586 | 0.638 | *Betaproteobacteria* | 5834 | 3260357 | *Dechloromonas aromatica RCB* |
| bin.1 | 94.47 | 5.398 | 0.639 | *Rhodocyclaceae* | 23904 | 3839115 | *Dechloromonas aromatica RCB* |
| bin.36 | 81.13 | 3.109 | 0.694 | *Proteobacteria* | 5824 | 3808482 | *Dechlorosoma suillum PS* |
| bin.13 | 87.02 | 3.903 | 0.665 | *Comamonadaceae* | 35222 | 6291176 | *Delftia acidovorans SPH-1* |
| bin.46 | 99.29 | 0.176 | 0.422 | *Flavobacteriaceae* | 290993 | 3678339 | *Flavobacterium johnsoniae UW101* |
| bin.14 | 92.28 | 4.761 | 0.316 | *Flavobacteriaceae* | 8076 | 3275348 | *Flavobacterium psychrophilum JIP02/86* |
| bin.3 | 87.25 | 4.87 | 0.324 | *Flavobacteriaceae* | 6009 | 2459718 | *Flavobacterium psychrophilum JIP02/86* |
| bin.76 | 98.7 | 7.476 | 0.324 | *Flavobacteriaceae* | 174805 | 3382773 | *Flavobacterium psychrophilum JIP02/86* |
| bin.99 | 70.42 | 3.418 | 0.522 | *Bacteria* | 2995 | 2223605 | *Gloeobacter violaceus PCC 7421* |
| bin.86 | 92.18 | 0.806 | 0.605 | *Deltaproteobacteria* | 24739 | 5390913 | *Haliangium ochraceum DSM 14365* |
| bin.81 | 84.65 | 5.856 | 0.688 | *Betaproteobacteria* | 16177 | 5845780 | *Herbaspirillum seropedicae SmR1* |
| bin.21 | 84.83 | 7.476 | 0.647 | *Burkholderiales* | 9982 | 4951863 | *Herbaspirillum seropedicae SmR1* |
| bin.10 | 93.62 | 0.467 | 0.486 | *Burkholderiales* | 15016 | 4711456 | *Janthinobacterium sp. Marseille* |
| bin.44 | 97.19 | 3.345 | 0.526 | *Burkholderiales* | 8816 | 4042228 | *Janthinobacterium sp. Marseille* |
| bin.42 | 83.76 | 5.229 | 0.486 | *Burkholderiales* | 7028 | 4054946 | *Janthinobacterium sp. Marseille* |
| bin.49 | 91.48 | 5.229 | 0.562 | *Bacteria* | 12968 | 5522534 | *Koribacter versatilis Ellin345* |
| bin.8 | 95.7 | 0.769 | 0.702 | *Actinomycetales* | 17668 | 2803529 | *Leifsonia xyli subsp. xyli str. CTCB07* |
| bin.85 | 86.5 | 3.829 | 0.72 | *Actinomycetales* | 5848 | 3008917 | *Leifsonia xyli subsp. xyli str. CTCB07* |
| bin.61 | 98.83 | 2.597 | 0.617 | *Alphaproteobacteria* | 63483 | 5131556 | *Magnetospirillum gryphiswaldense MSR-1* |
| bin.97 | 75.41 | 2.867 | 0.409 | *Bacteria* | 3051 | 1680797 | *Magnetospirillum gryphiswaldense MSR-1* |
| bin.79 | 89.27 | 3.337 | 0.647 | *Alphaproteobacteria* | 13387 | 3093664 | *Magnetospirillum magneticum AMB-1* |
| bin.83 | 74.21 | 6.304 | 0.662 | *Alphaproteobacteria* | 7443 | 3548405 | *Magnetospirillum magneticum AMB-1* |
| bin.27 | 89.71 | 7.485 | 0.631 | *Alphaproteobacteria* | 9925 | 2658184 | *Magnetospirillum magneticum AMB-1* |
| bin.78 | 99.5 | 2.269 | 0.634 | *Rhodospirillales* | 15791 | 4197061 | *Magnetospirillum magneticum AMB-1* |
| bin.34 | 90.04 | 2.985 | 0.649 | *Rhodospirillales* | 41036 | 3917248 | *Magnetospirillum magneticum AMB-1* |
| bin.18 | 83.72 | 7.779 | 0.565 | *Gammaproteobacteria* | 4807 | 3585117 | *Methylobacter tundripaludum SV96* |
| bin.52 | 93.88 | 2.985 | 0.62 | *Alphaproteobacteria* | 19416 | 2518930 | *Methylosinus trichosporium OB3b* |
| bin.20 | 95.51 | 3.159 | 0.669 | *Actinomycetales* | 28325 | 6102365 | *Mycobacterium avium 104* |
| bin.19 | 98.21 | 3.11 | 0.68 | *Actinomycetales* | 43314 | 6250628 | *Mycobacterium smegmatis str. MC2 155* |
| bin.82 | 78.47 | 3.68 | 0.708 | *Actinomycetales* | 4127 | 4610598 | *Mycobacterium sp. MCS* |
| bin.35 | 94.17 | 3.883 | 0.331 | *Archaea* | 18956 | 3654857 | *Nitrosopumilus maritimus SCM1* |
| bin.51 | 92.14 | 8.387 | 0.619 | *Betaproteobacteria* | 7804 | 2356574 | *Nitrosospira multiformis ATCC 25196* |
| bin.24 | 91.12 | 1.363 | 0.55 | *Bacteria* | 143599 | 4757366 | *Nitrospira defluvii* |
| bin.63 | 90.1 | 1.818 | 0.6 | *Bacteria* | 31346 | 2789718 | *Nitrospira defluvii* |
| bin.28 | 93.12 | 3.636 | 0.547 | *Bacteria* | 70367 | 4334193 | *Nitrospira defluvii* |
| bin.47 | 94.03 | 3.636 | 0.586 | *Bacteria* | 14707 | 3672658 | *Nitrospira defluvii* |
| bin.94 | 90.06 | 3.875 | 0.552 | *Bacteria* | 42731 | 4708955 | *Nitrospira defluvii* |
| bin.7 | 83.17 | 2.836 | 0.637 | *Sphingomonadales* | 8299 | 2175603 | *Novosphingobium aromaticivorans* |
| bin.54 | 82.54 | 3.773 | 0.636 | *Sphingomonadales* | 6480 | 2832311 | *Novosphingobium aromaticivorans* |
| bin.65 | 85.96 | 4.148 | 0.638 | *Sphingomonadales* | 8727 | 3326558 | *Novosphingobium aromaticivorans* |
| bin.38 | 94.7 | 1.351 | 0.427 | *Bacteria* | 17799 | 1281643 | *Parachlamydia sp. UWE25* |
| bin.72 | 80 | 5.202 | 0.634 | *Alphaproteobacteria* | 98689 | 4245456 | *Parvibaculum lavamentivorans DS-1* |
| bin.50 | 99.35 | 0 | 0.387 | *Bacteroidetes* | 189833 | 2153227 | *Pedobacter heparinus DSM 2366* |
| bin.56 | 95.21 | 0.015 | 0.385 | *Bacteroidetes* | 178978 | 4049945 | *Pedobacter heparinus DSM 2366* |
| bin.30 | 82.8 | 8.652 | 0.641 | *Bacteria* | 3456 | 7580545 | *Pelotomaculum thermopropionicum SI* |
| bin.6 | 74.14 | 3.051 | 0.639 | *Alphaproteobacteria* | 5058 | 2505049 | *Phenylobacterium zucineum HLK1* |
| bin.92 | 81.75 | 3.015 | 0.644 | *Burkholderiales* | 33677 | 3957638 | *Polaromonas sp. JS666* |
| bin.80 | 95.58 | 4.179 | 0.621 | *Pseudomonas* | 52950 | 6237036 | *Pseudomonas putida GB-1* |
| bin.58 | 84.67 | 4.811 | 0.631 | *Rhizobiales* | 14785 | 2860832 | *Rhodomicrobium vannielii ATCC 17100* |
| bin.16 | 99.56 | 1.739 | 0.618 | *Alphaproteobacteria* | 93135 | 3381203 | *Rhodospirillum rubrum* |
| bin.32 | 87.5 | 5.647 | 0.658 | *Alphaproteobacteria* | 14637 | 4974196 | *Rhodospirillum rubrum* |
| bin.64 | 94.9 | 1.824 | 0.608 | *Rhodospirillales* | 18132 | 2948988 | *Rhodospirillum rubrum* |
| bin.12 | 93.29 | 3.308 | 0.591 | *Rhodospirillales* | 53917 | 4065076 | *Rhodospirillum rubrum* |
| bin.98 | 92.1 | 9.59 | 0.592 | *Rhodospirillales* | 15395 | 4502992 | *Rhodospirillum rubrum* |
| bin.84 | 95.32 | 1.936 | 0.722 | *Actinomycetales* | 16557 | 3859912 | *Sanguibacter keddieii DSM 10542* |
| bin.66 | 81.31 | 4.171 | 0.619 | *Betaproteobacteria* | 7626 | 1899655 | *Sideroxydans lithotrophicus ES-1* |
| bin.77 | 78.12 | 4.42 | 0.71 | *Proteobacteria* | 4831 | 2504254 | *Sideroxydans lithotrophicus ES-1* |
| bin.73 | 98.26 | 0.869 | 0.565 | *Bacteria* | 120162 | 4912201 | *Solibacter usitatus Ellin6076* |
| bin.71 | 96.08 | 3.532 | 0.586 | *Bacteria* | 13709 | 5418868 | *Solibacter usitatus Ellin6076* |
| bin.68 | 73.61 | 2.782 | 0.655 | *Sphingomonadales* | 4018 | 2704412 | *Sphingobium japonicum UT26S* |
| bin.4 | 95.79 | 0.678 | 0.674 | *Alphaproteobacteria* | 71185 | 3335808 | *Sphingomonas wittichii RW1* |
| bin.2 | 93.21 | 2.756 | 0.66 | *Alphaproteobacteria* | 72646 | 4390633 | *Sphingomonas wittichii RW1* |
| bin.93 | 70.81 | 1.962 | 0.656 | *Sphingomonadales* | 5359 | 2187239 | *Sphingopyxis alaskensis RB2256* |
| bin.91 | 75.2 | 3.694 | 0.662 | *Sphingomonadales* | 4298 | 2626378 | *Sphingopyxis alaskensis RB2256* |
| bin.37 | 88.29 | 4.947 | 0.658 | *Sphingomonadales* | 14925 | 3738473 | *Sphingopyxis alaskensis RB2256* |
| bin.9 | 96.17 | 5.399 | 0.673 | *Sphingomonadales* | 44171 | 3565823 | *Sphingopyxis alaskensis RB2256* |
| bin.100 | 79.35 | 8.711 | 0.68 | *Sphingomonadales* | 7200 | 3100216 | *Sphingopyxis alaskensis RB2256* |
| bin.43 | 79.79 | 6.855 | 0.693 | *Xanthomonadaceae* | 6873 | 2296068 | *Stenotrophomonas maltophilia K279a* |
| bin.62 | 75.18 | 7.082 | 0.606 | *Rhodocyclaceae* | 4120 | 2391053 | *Thauera sp. MZ1T* |
| bin.89 | 77.83 | 7.056 | 0.658 | *Gammaproteobacteria* | 6322 | 3338458 | *Thioalkalivibrio sp. HL-EbGR7* |
| bin.41 | 73.89 | 8.764 | 0.642 | *Gammaproteobacteria* | 4788 | 3418343 | *Thioalkalivibrio sp. HL-EbGR7* |
| bin.22 | 98.97 | 3.129 | 0.338 | *Bacteria* | 15350 | 1921710 | *Thiomicrospira denitrificans ATCC 33889* |
| bin.95 | 70.16 | 2.997 | 0.694 | *Burkholderiales* | 59083 | 7345449 | *Variovorax paradoxus S110* |
| bin.45 | 83.36 | 4.33 | 0.697 | *Burkholderiales* | 17990 | 5562017 | *Variovorax paradoxus S110* |
| bin.74 | 94.05 | 2.162 | 0.637 | *Gammaproteobacteria* | 71311 | 3834733 | *Kangiella koreensis DSM 16069* |

Table S6. Genes related to resistance to antibiotics and toxic compounds within binned genomes. Annotation of genes using RAST/SEED.

| Bin No. | Closest neighbour assigned by RAST | Copper homeostasis | Cobalt-Zinc-Cadmium resistance | Resistance to Fluroquinolones | Copper homeostasis: Copper tolerance | Adaptation to d-cysteine | Aminoglyscoside adenylyltransferase | Tetracycline resistance and ribosomal protection type | Beta-lactamase | Resistance to vancomycin | Fosfomycin resistance | Zinc resistance | Strepthothricin resistance | Mercuric reductase | Mercury resistance operon | Arsenic resistance | Resistance to chromium compounds | Multidrug resistance efflux pumps | Mutidrug resistance, Tripartite system found in gram negatives | MexC-MexD-OprJ Multidrug Efflux System |
| --- | --- | --- | --- | --- | --- | --- | --- | --- | --- | --- | --- | --- | --- | --- | --- | --- | --- | --- | --- | --- |
| bin.15 | *Acidobacterium capsulatum ATCC 51196* | 7 | 15 | 5 | 4 | 1 | 0 | 0 | 6 | 0 | 0 | 22 | 0 | 0 | 0 | 0 | 1 | 0 | 0 | 0 |
| bin.55 | *Acidovorax avenae subsp. avenae ATCC 19860* | 14 | 18 | 4 | 3 | 0 | 0 | 0 | 5 | 0 | 0 | 0 | 0 | 0 | 0 | 0 | 3 | 16 | 0 | 0 |
| bin.29 | *Acidovorax avenae subsp. avenae ATCC 19860* | 9 | 16 | 0 | 2 | 0 | 0 | 0 | 8 | 0 | 0 | 1 | 0 | 0 | 0 | 0 | 3 | 17 | 3 | 0 |
| bin.69 | *Acidovorax radicis N35* | 3 | 31 | 0 | 4 | 0 | 0 | 0 | 5 | 0 | 0 | 0 | 0 | 0 | 0 | 0 | 2 | 13 | 3 | 0 |
| bin.48 | *Acidovorax radicis N35* | 16 | 36 | 4 | 3 | 0 | 0 | 0 | 4 | 0 | 0 | 1 | 0 | 1 | 4 | 4 | 2 | 21 | 3 | 0 |
| bin.70 | *Acidovorax radicis N35* | 8 | 22 | 0 | 4 | 0 | 0 | 0 | 4 | 0 | 0 | 0 | 0 | 1 | 0 | 0 | 2 | 17 | 3 | 0 |
| bin.53 | *Acinetobacter junii SH205* | 12 | 24 | 0 | 4 | 0 | 0 | 0 | 2 | 0 | 0 | 0 | 0 | 0 | 0 | 6 | 3 | 9 | 0 | 0 |
| bin.23 | *Acinetobacter lwoffii SH145* | 8 | 8 | 4 | 3 | 0 | 0 | 0 | 1 | 0 | 0 | 0 | 0 | 0 | 0 | 5 | 3 | 14 | 0 | 0 |
| bin.31 | *Acinetobacter sp. DR1* | 4 | 0 | 0 | 3 | 0 | 0 | 0 | 1 | 0 | 0 | 0 | 0 | 0 | 0 | 6 | 3 | 11 | 0 | 0 |
| bin.59 | *Alicycliphilus denitrificans BC* | 15 | 18 | 0 | 4 | 0 | 0 | 0 | 5 | 0 | 0 | 0 | 0 | 1 | 0 | 0 | 2 | 14 | 3 | 0 |
| bin.25 | *alpha proteobacterium BAL199* | 7 | 8 | 5 | 2 | 0 | 0 | 0 | 1 | 0 | 0 | 0 | 0 | 1 | 1 | 4 | 1 | 0 | 0 | 0 |
| bin.26 | *Azospirillum sp. B510* | 3 | 3 | 4 | 2 | 0 | 0 | 0 | 1 | 1 | 1 | 0 | 0 | 1 | 1 | 0 | 0 | 5 | 0 | 0 |
| bin.5 | *Azospirillum sp. B510* | 13 | 18 | 3 | 3 | 3 | 0 | 0 | 9 | 0 | 0 | 0 | 0 | 2 | 3 | 7 | 3 | 15 | 0 | 0 |
| bin.57 | *Brevundimonas sp. BAL3* | 4 | 9 | 3 | 2 | 0 | 0 | 0 | 5 | 0 | 0 | 0 | 0 | 0 | 0 | 2 | 2 | 8 | 0 | 0 |
| bin.101 | *Brevundimonas sp. BAL3* | 39 | 46 | 4 | 3 | 0 | 0 | 0 | 8 | 0 | 0 | 0 | 0 | 2 | 4 | 11 | 1 | 10 | 0 | 0 |
| bin.60 | *Caulobacter sp. K31* | 21 | 10 | 2 | 2 | 0 | 0 | 0 | 8 | 0 | 0 | 1 | 0 | 1 | 3 | 0 | 0 | 0 | 0 | 0 |
| bin.87 | *Chitinophaga pinensis DSM 2588* | 3 | 6 | 4 | 0 | 0 | 0 | 0 | 8 | 0 | 0 | 2 | 0 | 0 | 0 | 0 | 0 | 7 | 3 | 0 |
| bin.75 | *Chitinophaga pinensis DSM 2588* | 6 | 24 | 4 | 0 | 0 | 0 | 0 | 7 | 0 | 0 | 2 | 0 | 1 | 0 | 3 | 0 | 18 | 3 | 0 |
| bin.88 | *Chitinophaga pinensis DSM 2588* | 4 | 12 | 4 | 0 | 0 | 0 | 0 | 7 | 0 | 0 | 1 | 0 | 0 | 0 | 0 | 0 | 6 | 4 | 0 |
| bin.39 | *Chromobacterium violaceum ATCC 12472* | 2 | 11 | 0 | 4 | 0 | 0 | 0 | 0 | 0 | 0 | 1 | 0 | 0 | 0 | 1 | 2 | 14 | 0 | 0 |
| bin.40 | *Chromobacterium violaceum ATCC 12472* | 12 | 20 | 0 | 0 | 0 | 0 | 0 | 1 | 0 | 0 | 1 | 0 | 0 | 0 | 10 | 3 | 18 | 9 | 0 |
| bin.33 | *Chryseobacterium gleum ATCC 35910* | 5 | 17 | 4 | 0 | 0 | 0 | 0 | 10 | 1 | 0 | 2 | 1 | 0 | 0 | 5 | 0 | 0 | 8 | 0 |
| bin.11 | *Comamonas testosteroni KF-1* | 9 | 6 | 0 | 3 | 0 | 0 | 0 | 0 | 0 | 0 | 0 | 0 | 0 | 0 | 3 | 0 | 17 | 3 | 0 |
| bin.102 | *Comamonas testosteroni KF-1* | 14 | 58 | 3 | 3 | 0 | 0 | 0 | 3 | 0 | 0 | 2 | 0 | 0 | 0 | 6 | 4 | 19 | 6 | 1 |
| bin.90 | *Conexibacter woesei DSM 14684* | 15 | 4 | 2 | 0 | 1 | 0 | 0 | 3 | 2 | 0 | 0 | 0 | 2 | 0 | 7 | 1 | 0 | 0 | 0 |
| bin.17 | *Cupriavidus metallidurans CH34* | 4 | 27 | 4 | 5 | 0 | 0 | 0 | 1 | 0 | 0 | 0 | 0 | 0 | 0 | 0 | 3 | 15 | 0 | 0 |
| bin.67 | *Dechloromonas aromatica RCB* | 8 | 34 | 6 | 6 | 0 | 0 | 0 | 3 | 0 | 0 | 0 | 0 | 0 | 0 | 3 | 2 | 17 | 0 | 0 |
| bin.1 | *Dechloromonas aromatica RCB* | 11 | 30 | 4 | 3 | 0 | 0 | 0 | 1 | 0 | 0 | 0 | 0 | 0 | 0 | 2 | 1 | 12 | 0 | 0 |
| bin.36 | *Dechlorosoma suillum PS* | 11 | 32 | 4 | 2 | 0 | 0 | 0 | 2 | 0 | 0 | 0 | 0 | 1 | 0 | 0 | 3 | 11 | 0 | 0 |
| bin.13 | *Delftia acidovorans SPH-1* | 16 | 48 | 2 | 5 | 0 | 0 | 0 | 8 | 0 | 0 | 3 | 0 | 0 | 0 | 8 | 4 | 34 | 6 | 2 |
| bin.46 | *Flavobacterium johnsoniae UW101* | 3 | 29 | 4 | 0 | 0 | 0 | 0 | 7 | 0 | 0 | 1 | 0 | 0 | 0 | 0 | 0 | 10 | 9 | 0 |
| bin.14 | *Flavobacterium psychrophilum JIP02/86* | 2 | 19 | 2 | 0 | 0 | 0 | 0 | 7 | 0 | 0 | 0 | 0 | 0 | 0 | 0 | 0 | 0 | 3 | 0 |
| bin.3 | *Flavobacterium psychrophilum JIP02/86* | 3 | 16 | 5 | 0 | 0 | 0 | 0 | 2 | 0 | 0 | 0 | 0 | 0 | 0 | 4 | 0 | 0 | 0 | 0 |
| bin.76 | *Flavobacterium psychrophilum JIP02/86* | 4 | 13 | 5 | 0 | 0 | 0 | 3 | 6 | 0 | 0 | 0 | 0 | 0 | 0 | 0 | 0 | 0 | 6 | 0 |
| bin.99 | *Gloeobacter violaceus PCC 7421* | 1 | 5 | 2 | 0 | 0 | 0 | 0 | 0 | 1 | 0 | 0 | 0 | 0 | 0 | 0 | 0 | 0 | 0 | 0 |
| bin.86 | *Haliangium ochraceum DSM 14365* | 6 | 21 | 4 | 4 | 0 | 0 | 0 | 4 | 1 | 0 | 27 | 0 | 2 | 2 | 6 | 0 | 0 | 0 | 0 |
| bin.81 | *Herbaspirillum seropedicae SmR1* | 9 | 13 | 4 | 5 | 0 | 0 | 0 | 2 | 0 | 0 | 0 | 0 | 0 | 0 | 0 | 4 | 17 | 3 | 0 |
| bin.21 | *Herbaspirillum seropedicae SmR1* | 12 | 22 | 4 | 4 | 0 | 0 | 0 | 6 | 0 | 0 | 1 | 0 | 0 | 0 | 0 | 1 | 20 | 0 | 0 |
| bin.10 | *Janthinobacterium sp. Marseille* | 7 | 22 | 4 | 6 | 0 | 0 | 0 | 6 | 0 | 1 | 0 | 0 | 0 | 0 | 0 | 1 | 22 | 3 | 0 |
| bin.44 | *Janthinobacterium sp. Marseille* | 5 | 21 | 4 | 4 | 0 | 0 | 0 | 3 | 0 | 0 | 0 | 0 | 0 | 0 | 0 | 1 | 14 | 3 | 0 |
| bin.42 | *Janthinobacterium sp. Marseille* | 2 | 27 | 4 | 0 | 0 | 1 | 0 | 10 | 0 | 0 | 0 | 0 | 0 | 0 | 1 | 2 | 13 | 0 | 0 |
| bin.49 | *Koribacter versatilis Ellin345* | 5 | 46 | 2 | 3 | 0 | 0 | 0 | 14 | 0 | 0 | 9 | 0 | 1 | 0 | 3 | 1 | 12 | 13 | 0 |
| bin.8 | *Leifsonia xyli subsp. xyli str. CTCB07* | 10 | 4 | 4 | 0 | 0 | 0 | 0 | 0 | 0 | 0 | 0 | 0 | 2 | 1 | 7 | 1 | 0 | 0 | 0 |
| bin.85 | *Leifsonia xyli subsp. xyli str. CTCB07* | 8 | 2 | 0 | 0 | 0 | 0 | 0 | 5 | 0 | 0 | 0 | 0 | 1 | 0 | 3 | 0 | 0 | 0 | 0 |
| bin.61 | *Magnetospirillum gryphiswaldense MSR-1* | 15 | 28 | 4 | 3 | 0 | 0 | 0 | 6 | 0 | 0 | 1 | 0 | 0 | 0 | 10 | 3 | 0 | 0 | 0 |
| bin.97 | *Magnetospirillum gryphiswaldense MSR-1* | 2 | 4 | 3 | 4 | 0 | 0 | 0 | 0 | 0 | 0 | 0 | 0 | 0 | 0 | 0 | 0 | 4 | 0 | 0 |
| bin.79 | *Magnetospirillum magneticum AMB-1* | 18 | 14 | 0 | 0 | 0 | 0 | 0 | 3 | 0 | 0 | 1 | 0 | 0 | 0 | 4 | 1 | 13 | 0 | 0 |
| bin.83 | *Magnetospirillum magneticum AMB-1* | 12 | 7 | 4 | 2 | 0 | 0 | 0 | 4 | 0 | 0 | 0 | 0 | 1 | 1 | 3 | 6 | 9 | 0 | 0 |
| bin.27 | *Magnetospirillum magneticum AMB-1* | 12 | 20 | 4 | 0 | 0 | 0 | 1 | 2 | 0 | 0 | 0 | 0 | 0 | 0 | 0 | 1 | 0 | 4 | 0 |
| bin.78 | *Magnetospirillum magneticum AMB-1* | 18 | 14 | 0 | 0 | 0 | 0 | 0 | 3 | 0 | 0 | 1 | 0 | 0 | 0 | 4 | 1 | 13 | 0 | 0 |
| bin.34 | *Magnetospirillum magneticum AMB-1* | 10 | 24 | 4 | 3 | 0 | 0 | 0 | 11 | 0 | 0 | 1 | 0 | 1 | 0 | 0 | 1 | 0 | 5 | 0 |
| bin.18 | *Methylobacter tundripaludum SV96* | 15 | 30 | 0 | 5 | 0 | 0 | 1 | 2 | 0 | 0 | 2 | 0 | 1 | 0 | 6 | 2 | 15 | 0 | 0 |
| bin.52 | *Methylosinus trichosporium OB3b* | 9 | 28 | 4 | 3 | 0 | 0 | 0 | 3 | 0 | 0 | 0 | 0 | 0 | 0 | 0 | 1 | 8 | 6 | 0 |
| bin.20 | *Mycobacterium avium 104* | 8 | 2 | 2 | 0 | 0 | 0 | 0 | 9 | 0 | 0 | 0 | 0 | 4 | 1 | 8 | 0 | 0 | 0 | 0 |
| bin.19 | *Mycobacterium smegmatis str. MC2 155* | 11 | 9 | 2 | 0 | 0 | 0 | 0 | 9 | 0 | 0 | 0 | 0 | 1 | 0 | 10 | 0 | 0 | 0 | 0 |
| bin.82 | *Mycobacterium sp. MCS* | 14 | 7 | 0 | 0 | 0 | 0 | 0 | 6 | 0 | 0 | 0 | 0 | 2 | 1 | 8 | 0 | 0 | 0 | 0 |
| bin.35 | *Nitrosopumilus maritimus SCM1* | 32 | 2 | 0 | 0 | 0 | 0 | 0 | 4 | 0 | 0 | 0 | 0 | 1 | 1 | 4 | 0 | 0 | 0 | 0 |
| bin.51 | *Nitrosospira multiformis ATCC 25196* | 8 | 22 | 2 | 0 | 0 | 0 | 0 | 2 | 0 | 0 | 0 | 0 | 1 | 1 | 4 | 0 | 0 | 3 | 0 |
| bin.24 | *Nitrospira defluvii* | 17 | 60 | 2 | 3 | 0 | 0 | 0 | 2 | 0 | 0 | 19 | 0 | 4 | 2 | 4 | 0 | 13 | 0 | 0 |
| bin.63 | *Nitrospira defluvii* | 7 | 36 | 1 | 4 | 0 | 0 | 0 | 3 | 0 | 0 | 14 | 0 | 2 | 1 | 0 | 0 | 9 | 0 | 0 |
| bin.28 | *Nitrospira defluvii* | 16 | 39 | 2 | 3 | 0 | 0 | 0 | 2 | 0 | 0 | 21 | 0 | 2 | 2 | 0 | 0 | 17 | 0 | 0 |
| bin.47 | *Nitrospira defluvii* | 13 | 49 | 2 | 3 | 0 | 0 | 0 | 2 | 0 | 0 | 25 | 0 | 3 | 1 | 3 | 0 | 0 | 0 | 0 |
| bin.94 | *Nitrospira defluvii* | 15 | 69 | 3 | 3 | 0 | 0 | 0 | 5 | 0 | 0 | 21 | 0 | 4 | 3 | 0 | 0 | 20 | 0 | 0 |
| bin.7 | *Novosphingobium aromaticivorans* | 10 | 12 | 3 | 3 | 0 | 0 | 0 | 5 | 0 | 0 | 2 | 0 | 0 | 0 | 0 | 1 | 0 | 6 | 0 |
| bin.54 | *Novosphingobium aromaticivorans* | 3 | 5 | 3 | 0 | 0 | 0 | 0 | 9 | 0 | 0 | 0 | 0 | 1 | 0 | 4 | 1 | 0 | 4 | 0 |
| bin.65 | *Novosphingobium aromaticivorans* | 14 | 9 | 3 | 3 | 0 | 0 | 0 | 7 | 0 | 0 | 0 | 0 | 0 | 0 | 8 | 1 | 8 | 3 | 0 |
| bin.38 | *Parachlamydia sp. UWE25* | 1 | 0 | 2 | 0 | 0 | 0 | 0 | 3 | 0 | 0 | 1 | 0 | 0 | 0 | 0 | 0 | 0 | 3 | 0 |
| bin.72 | *Parvibaculum lavamentivorans DS-1* | 13 | 30 | 4 | 0 | 0 | 0 | 0 | 23 | 1 | 0 | 1 | 0 | 0 | 0 | 0 | 1 | 17 | 0 | 0 |
| bin.50 | *Pedobacter heparinus DSM 2366* | 3 | 10 | 4 | 0 | 0 | 0 | 0 | 3 | 0 | 0 | 1 | 0 | 0 | 0 | 0 | 0 | 0 | 0 | 0 |
| bin.56 | *Pedobacter heparinus DSM 2366* | 4 | 29 | 4 | 0 | 0 | 0 | 0 | 8 | 0 | 0 | 2 | 0 | 0 | 0 | 4 | 2 | 0 | 3 | 0 |
| bin.30 | *Pelotomaculum thermopropionicum SI* | 10 | 27 | 2 | 2 | 0 | 0 | 0 | 22 | 4 | 0 | 0 | 0 | 1 | 0 | 0 | 2 | 5 | 0 | 0 |
| bin.6 | *Phenylobacterium zucineum HLK1* | 13 | 17 | 0 | 3 | 0 | 0 | 0 | 5 | 0 | 0 | 0 | 0 | 0 | 0 | 0 | 0 | 0 | 9 | 0 |
| bin.92 | *Polaromonas sp. JS666* | 12 | 29 | 0 | 3 | 0 | 0 | 0 | 1 | 0 | 0 | 0 | 0 | 0 | 0 | 0 | 1 | 14 | 3 | 0 |
| bin.80 | *Pseudomonas putida GB-1* | 39 | 51 | 3 | 3 | 0 | 0 | 0 | 3 | 0 | 0 | 3 | 0 | 3 | 6 | 14 | 4 | 15 | 15 | 4 |
| bin.58 | *Rhodomicrobium vannielii ATCC 17100* | 13 | 14 | 3 | 3 | 0 | 0 | 0 | 2 | 0 | 0 | 0 | 0 | 1 | 1 | 0 | 1 | 5 | 0 | 0 |
| bin.16 | *Rhodospirillum rubrum* | 18 | 13 | 4 | 4 | 0 | 0 | 0 | 3 | 1 | 0 | 1 | 0 | 0 | 0 | 6 | 2 | 11 | 0 | 0 |
| bin.32 | *Rhodospirillum rubrum* | 15 | 12 | 4 | 2 | 0 | 0 | 0 | 3 | 1 | 0 | 1 | 0 | 0 | 0 | 6 | 2 | 0 | 0 | 0 |
| bin.64 | *Rhodospirillum rubrum* | 0 | 18 | 4 | 3 | 0 | 0 | 0 | 3 | 0 | 0 | 1 | 0 | 0 | 0 | 0 | 2 | 0 | 0 | 0 |
| bin.12 | *Rhodospirillum rubrum* | 8 | 48 | 4 | 3 | 0 | 0 | 0 | 4 | 0 | 0 | 2 | 0 | 0 | 0 | 0 | 1 | 0 | 0 | 0 |
| bin.98 | *Rhodospirillum rubrum* | 11 | 40 | 4 | 3 | 0 | 0 | 0 | 4 | 0 | 0 | 1 | 0 | 1 | 1 | 0 | 3 | 0 | 0 | 0 |
| bin.84 | *Sanguibacter keddieii DSM 10542* | 9 | 7 | 4 | 0 | 0 | 0 | 0 | 5 | 1 | 0 | 0 | 0 | 1 | 1 | 7 | 0 | 0 | 0 | 0 |
| bin.66 | *Sideroxydans lithotrophicus ES-1* | 10 | 10 | 2 | 3 | 0 | 0 | 0 | 2 | 0 | 0 | 1 | 0 | 0 | 0 | 3 | 0 | 0 | 0 | 0 |
| bin.77 | *Sideroxydans lithotrophicus ES-1* | 3 | 9 | 3 | 3 | 0 | 0 | 0 | 1 | 0 | 0 | 0 | 0 | 2 | 0 | 0 | 2 | 0 | 0 | 0 |
| bin.73 | *Solibacter usitatus Ellin6076* | 21 | 74 | 2 | 4 | 0 | 0 | 0 | 6 | 0 | 0 | 8 | 0 | 1 | 0 | 0 | 4 | 6 | 4 | 0 |
| bin.71 | *Solibacter usitatus Ellin6076* | 19 | 82 | 2 | 4 | 0 | 0 | 0 | 4 | 0 | 0 | 17 | 0 | 3 | 0 | 0 | 3 | 9 | 5 | 0 |
| bin.68 | *Sphingobium japonicum UT26S* | 5 | 12 | 2 | 0 | 0 | 0 | 0 | 6 | 0 | 0 | 1 | 0 | 0 | 0 | 0 | 0 | 13 | 0 | 0 |
| bin.4 | *Sphingomonas wittichii RW1* | 9 | 20 | 4 | 3 | 0 | 0 | 0 | 3 | 0 | 0 | 0 | 0 | 1 | 1 | 3 | 1 | 10 | 10 | 0 |
| bin.2 | *Sphingomonas wittichii RW1* | 15 | 18 | 5 | 4 | 0 | 0 | 0 | 8 | 0 | 0 | 0 | 0 | 0 | 0 | 6 | 1 | 16 | 6 | 0 |
| bin.93 | *Sphingopyxis alaskensis RB2256* | 4 | 20 | 3 | 0 | 0 | 0 | 0 | 10 | 0 | 0 | 0 | 0 | 0 | 0 | 0 | 0 | 14 | 0 | 0 |
| bin.91 | *Sphingopyxis alaskensis RB2256* | 6 | 8 | 4 | 0 | 0 | 0 | 0 | 8 | 0 | 0 | 0 | 0 | 0 | 0 | 0 | 1 | 10 | 11 | 0 |
| bin.37 | *Sphingopyxis alaskensis RB2256* | 7 | 22 | 5 | 4 | 0 | 0 | 0 | 8 | 0 | 0 | 0 | 0 | 0 | 0 | 4 | 4 | 25 | 5 | 0 |
| bin.9 | *Sphingopyxis alaskensis RB2256* | 16 | 32 | 3 | 3 | 0 | 0 | 0 | 8 | 0 | 0 | 0 | 0 | 0 | 0 | 9 | 2 | 20 | 8 | 0 |
| bin.100 | *Sphingopyxis alaskensis RB2256* | 3 | 20 | 5 | 3 | 1 | 0 | 0 | 10 | 0 | 0 | 1 | 0 | 0 | 0 | 0 | 0 | 15 | 0 | 0 |
| bin.43 | *Stenotrophomonas maltophilia K279a* | 3 | 6 | 4 | 0 | 0 | 0 | 0 | 2 | 0 | 0 | 0 | 0 | 0 | 0 | 0 | 1 | 10 | 3 | 0 |
| bin.62 | *Thauera sp. MZ1T* | 5 | 23 | 0 | 2 | 0 | 0 | 0 | 0 | 0 | 0 | 1 | 0 | 0 | 0 | 0 | 0 | 7 | 0 | 0 |
| bin.89 | *Thioalkalivibrio sp. HL-EbGR7* | 14 | 21 | 5 | 0 | 0 | 0 | 0 | 1 | 0 | 0 | 0 | 0 | 1 | 0 | 0 | 0 | 12 | 0 | 0 |
| bin.41 | *Thioalkalivibrio sp. HL-EbGR7* | 6 | 31 | 3 | 3 | 0 | 0 | 0 | 2 | 0 | 0 | 0 | 0 | 0 | 0 | 0 | 1 | 15 | 12 | 0 |
| bin.22 | *Thiomicrospira denitrificans ATCC 33889* | 3 | 8 | 2 | 0 | 0 | 0 | 0 | 1 | 0 | 0 | 0 | 0 | 0 | 0 | 4 | 0 | 18 | 0 | 0 |
| bin.95 | *Variovorax paradoxus S110* | 22 | 54 | 3 | 4 | 0 | 0 | 2 | 10 | 1 | 0 | 2 | 0 | 0 | 0 | 5 | 3 | 29 | 10 | 0 |
| bin.45 | *Variovorax paradoxus S110* | 13 | 29 | 3 | 5 | 0 | 0 | 0 | 5 | 0 | 0 | 1 | 0 | 0 | 0 | 5 | 2 | 23 | 5 | 0 |
| bin.74 | *Kangiella koreensis DSM 16069* | 12 | 23 | 4 | 4 | 0 | 0 | 0 | 3 | 0 | 0 | 0 | 0 | 0 | 0 | 0 | 1 | 24 | 0 | 0 |
